# Time-Frequency Feature Extraction and Modal Component Reconstruction for Structural Dynamic Monitoring Using MTM-eNTFT

**DOI:** 10.3390/e28091001

**Published:** 2026-09-07

**Authors:** Ling’ai Li, Junwei Wang, Chi Zhang

**Affiliations:** 1 School of Civil Engineering, Sichuan University of Science & Engineering, Zigong 643002, China; 2 School of Geodesy and Geomatics, Wuhan University, Wuhan 430072, China; 3 Chongqing Sichuang Road and Bridge Engineering Co., Ltd., Chongqing 400064, China; 4 School of Civil and Hydraulic Engineering, Chongqing University of Science and Technology, Chongqing 401331, China

**Keywords:** time-frequency feature extraction, structural dynamic monitoring, multitaper spectral estimation, normal time-frequency transform, multiscale permutation entropy

## Abstract

Field-measured structural responses are often noisy, multicomponent, nonstationary, and finite in length, complicating dominant-frequency identification, component extraction, and time-frequency characterization. This study develops an MTM-assisted Normal Time-Frequency Transform procedure with multiscale permutation entropy (MPE)-guided endpoint extension, termed MTM-eNTFT, to improve target-frequency-band determination and mitigate boundary-related reconstruction errors. Multitaper spectral estimation is used to determine stable target-frequency regions, while MPE-guided endpoint extension is used before band-limited NTFT reconstruction. Under the investigated simulation conditions, MTM-eNTFT provides more accurate component reconstruction, better noise suppression, and smaller boundary-related reconstruction errors than conventional NTFT, CEEMDAN, VMD, and SET. The reconstructed signal yields an RMSE below 0.08, a Pearson correlation coefficient over 0.98, and an SNR improvement of about 19 dB relative to the noisy input. The method is also applied to a selected continuous 15-min X-direction acceleration record acquired at a roof-corner sensor of a 68-storey building in Hong Kong during a high-wind event. Two dominant frequency components centered at approximately 0.208 and 0.965 Hz are extracted. Their energy increases between approximately 130 and 460 s, possibly indicating a temporary increase in the measured dynamic response. The results indicate the applicability of MTM-eNTFT to component extraction and time-frequency characterization of noisy finite-length structural-response records.

## 1. Introduction

Large civil structures exhibit complex dynamic responses under wind, seismic, traffic, and other environmental or operational excitations. Measured displacement and acceleration responses provide essential information on dominant frequencies, vibration amplitudes, time-varying energy distributions, and modal characteristics, which are fundamental to structural identification, serviceability evaluation, and safety assessment. With the increasing deployment of long-term monitoring systems, reliable dynamic-feature extraction from field measurements has become important for large civil infrastructure under variable operating conditions [1,2,3].

Nevertheless, field-measured structural responses are often noisy, multimodal, nonstationary, and finite in length. Closely spaced modal components, locally coupled vibrations, and transient disturbances may overlap in the time-frequency domain, complicating modal-frequency identification, component separation, and time-varying energy characterization. Noise, operational variability, and limited observation duration further reduce the reliability of these estimates [4,5,6]. In addition, the lack of valid data outside the record boundaries may cause edge effects in time-frequency analysis and component reconstruction. These challenges are amplified when the objective is to extract individual dynamic components from short or noisy monitoring records.

Various signal-processing methods have been applied to nonstationary structural-response analysis. Fourier transform (FT) provides global spectral information but cannot characterize temporal variations, whereas short-time Fourier transform (STFT) and wavelet transform (WT) offer localized or multiresolution representations at the cost of dependence on window, basis, or scale selection [7,8,9]. Hilbert–Huang transform (HHT) provides adaptive decomposition, but its empirical mode decomposition procedure may suffer from mode mixing, noise sensitivity, and endpoint effects [10,11]. Variational mode decomposition (VMD) and synchroextracting transform (SET) have been applied to nonstationary structural response analysis, but their performance depends on parameter selection and noise sensitivity [12,13,14,15]. Complementary Ensemble Empirical Mode Decomposition with Adaptive Noise (CEEMDAN) alleviates mode mixing to some extent, but residual noise and endpoint effects remain pronounced, and the decomposition quality is sensitive to algorithmic parameters such as noise amplitude and ensemble size [16,17,18].

The Normal Time-Frequency Transform (NTFT) is suitable for analyzing time-varying harmonic components within prescribed frequency ranges. With appropriate kernel and band settings, it provides time-frequency characterization while preserving the physical dimension of the analyzed signal, enabling the estimation of instantaneous amplitude, frequency, and phase [19,20]. However, NTFT-based separation depends on appropriate target bands. An excessively broad band may retain neighboring modes and noise, whereas an excessively narrow band may omit part of the target component. In addition, the finite support of the transform kernel can produce coefficient and reconstruction errors near the signal boundaries [21,22]. Multitaper spectral estimation (MTM) uses multiple orthogonal tapers to reduce spectral leakage and spectral-estimation variance, making it useful for identifying dominant frequency regions in noisy finite-length records [23,24]. It can therefore provide data-informed frequency priors for band-limited NTFT analysis, although it does not address boundary-induced reconstruction errors. Conversely, conventional signal-extension techniques—including zero-padding, mirror or periodic extension, polynomial extrapolation, and autoregressive prediction—may introduce discontinuity, phase, or amplitude errors when applied to noisy multicomponent signals [25,26]. Crucially, frequency-band identification and endpoint compensation are often treated as separate steps, and their coordinated integration into NTFT-based reconstruction has not been systematically addressed.

To address these issues, this study proposes MTM-eNTFT, an NTFT-based method for time-frequency feature extraction and modal reconstruction of noisy finite-length structural signals. Rather than treating spectral identification and boundary treatment as independent modules, the proposed framework couples MTM-based frequency-prior estimation with MPE-constrained endpoint extension within a band-limited NTFT reconstruction procedure. MTM provides robust frequency priors that guide the definition of modal bands and also constrain the allowable extension range through the dominant modal period. MPE then selects reference segments based on multiscale dynamical complexity and waveform similarity, while the extension length is jointly determined by the edge-effect length and the periodicity constraint derived from MTM. This coupling embeds physical periodicity information into the entropy-based matching process, enabling NTFT to operate under explicit frequency priors and boundary-aware signal extension—two aspects that are not simultaneously addressed in conventional implementations. The effectiveness of the proposed framework is validated using numerical simulations and field acceleration data from a 68-floor super-tall building in Hong Kong during a high-wind event, with comparative evaluations against conventional NTFT, VMD, and SET.

## 2. Theoretical Bases of MTM-eNTFT

### 2.1. Theoretical Bases of Normal Time-Frequency Transform

NTFT is an effective method for extracting features from non-stationary signals, which can provide a standard time-frequency filtering tool because its inverse transformation is independent of the kernel function (Equation (4)). The detailed definition of the NTFT method can be found in previous research [19]. A harmonic signal can be defined as:

(1) $$f(t)=A\exp(i\beta \;t)=\;\left|A\right|\;\exp\;(i\;(\beta t+\varphi ))$$

where $\left|A\right|$, β and φ denote the amplitude, instantaneous frequency and initial phase, respectively; βt+φ denotes the instantaneous phase. The NTFT of harmonic signal f(t) is defined as:

(2) $$\Psi f(\tau ,\varpi )=\int_{R}f(t)\bar{\psi }(t-\tau ,\varpi )d_{t}\;,\;\tau ,\;\varpi \in R$$

where ψ(t−τ,ϖ) denotes the kernel function; “–” means taking the conjugate; τ,ϖ represents the time and frequency indices, respectively. The kernel function must meet the following Equation (3).

(3) $$\psi (\omega ,\varpi )=\max\left|\overset{⌢}{\psi }(\omega ,\varpi )\right|=1\Leftrightarrow \omega =\varpi$$

where Equation (3) illustrates that the maximum of the kernel function is obtained only when the frequency index is equal to the instantaneous frequency.

The typical NTFT kernel function can be constructed as:

(4) $$\psi (t,\varpi )=\left|\mu (\varpi )\right|w(\mu (\varpi )t)\exp(i\varpi t),w(t)\in \Omega (R),(\mu (\varpi )\in R)\overset{•}{\neq }0$$

where μ(ϖ) represents the time-frequency Resolution Adaptor (TFRA). Since μ(ϖ) has no effect on the inverse transformation of NTFT and does not affect the accuracy of recognizing signal time-frequency features, μ(ϖ) can be adaptively designed based on the time-frequency structure characteristics of the signal and specific time-frequency resolution requirements. Letting μ(ϖ)=1 or μ(ϖ)=ϖ yields two special NTFT forms (i.e., the Normal Gabor transform (GT) and the Normal Morlet Wavelet transform (NMWT)). Other mathematical expressions and any other constants except zero can also be used to construct a TFRA. In this article, the value of μ(ϖ) is set to ϖ. The dot above the inequality sign means “almost anywhere”; w(t) denotes the normal window function, and Ω(R) represents the set of all normal windows. Commonly used window functions include Gaussian, Hanning, Hamming, Blackman, Black-Harris, and Nuttal windows, etc. The normal Gaussian window function is a good choice and it satisfied with general time-frequency analysis requirements in NTFT.

The inverse transform of the NTFT can be expressed as follows:

(5) $$f(t)=\frac{1}{2\pi }\int_{R}\int_{R}\psi f(\tau ,\varpi )\exp(i\varpi (t-\tau ))d\tau d\varpi$$

### 2.2. Limitations of NTFT and Motivation for the MTM-eNTFT Method

#### 2.2.1. Problems of NTFT Extraction for Multimodal Signals Without Band Division

NTFT is capable of characterizing the local time-frequency features of non-stationary signals. However, for multimodal signals commonly encountered in structural dynamic monitoring, if NTFT is directly applied to the original signal as a whole without prior band division for the target mode, the energy distributions of different modal components in the time-frequency plane tend to become coupled and overlapped, thereby degrading the extraction accuracy of the target component. This issue is particularly pronounced when the target component has a relatively low amplitude, under which condition its effective time-frequency features become even more difficult to identify in the presence of background noise.

**Figure 1:**
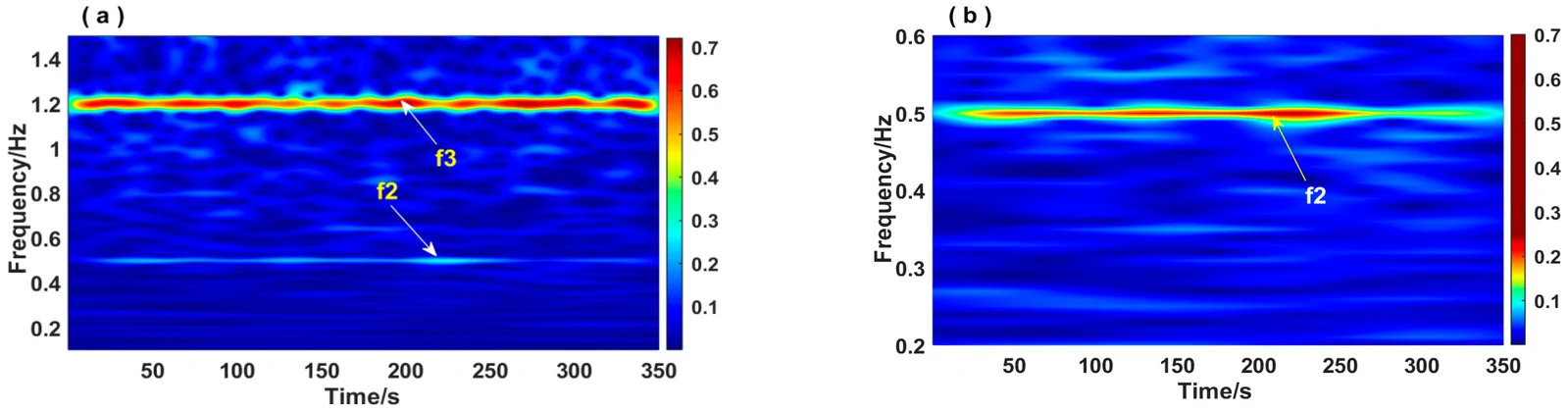
Time-frequency spectrum of the composite simulated signal. (**a**) Full-band: weak target obscured; (**b**) Band-divided: weak target enhanced and clear.

To illustrate this phenomenon, we construct a multimodal simulated signal with a sampling frequency of 10 Hz and a sampling duration of 360 s. The signal components have frequencies of 0.5 Hz, 0.8 Hz, and 1.2 Hz, respectively, and Gaussian white noise with a signal-to-noise ratio of −2.5 dB is added to the simulated signal. Figure 1 shows its NTFT time-frequency spectrum. The two subplots focus on different local frequency bands to better illustrate the effect of band-wise processing on weak signal identification. It can be observed that, in the spectrum without band division (Figure 1a), the time-frequency signature of target component 2 is relatively obscure. This is because the amplitude of this component is comparatively low, and its effective energy can be readily masked by background noise as well as other stronger components under full-band analysis, causing the corresponding time-frequency ridge to become attenuated and even locally indistinct. By contrast, Figure 1b presents the time-frequency spectrum after band division for component 2. In this case, the energy distribution becomes significantly more concentrated, the target trajectory is more clearly identifiable, and the interference from non-target components is markedly reduced. These results indicate that, for multimodal signals, the effective application of NTFT relies first on reliable prior frequency information and appropriate band constraints; otherwise, both the extraction accuracy and robustness of weak modal components are difficult to guarantee.

#### 2.2.2. Insufficient Extraction Accuracy Induced by Edge Effects

In addition to the issue of band division, NTFT is also affected by edge effects when processing finite-length signals. The implementation of NTFT relies on the sliding operation of a local analysis window or kernel function along the time axis. When the analysis window approaches the beginning or the end of the signal, complete data support cannot be obtained, and the time-frequency representation in the boundary region is therefore subject to truncation. Such incomplete local information distorts the time-frequency energy distribution near the endpoints, forming an “edge-effect” region and consequently impairing the extraction accuracy of signal components in the initial and terminal segments.

**Figure 2:**
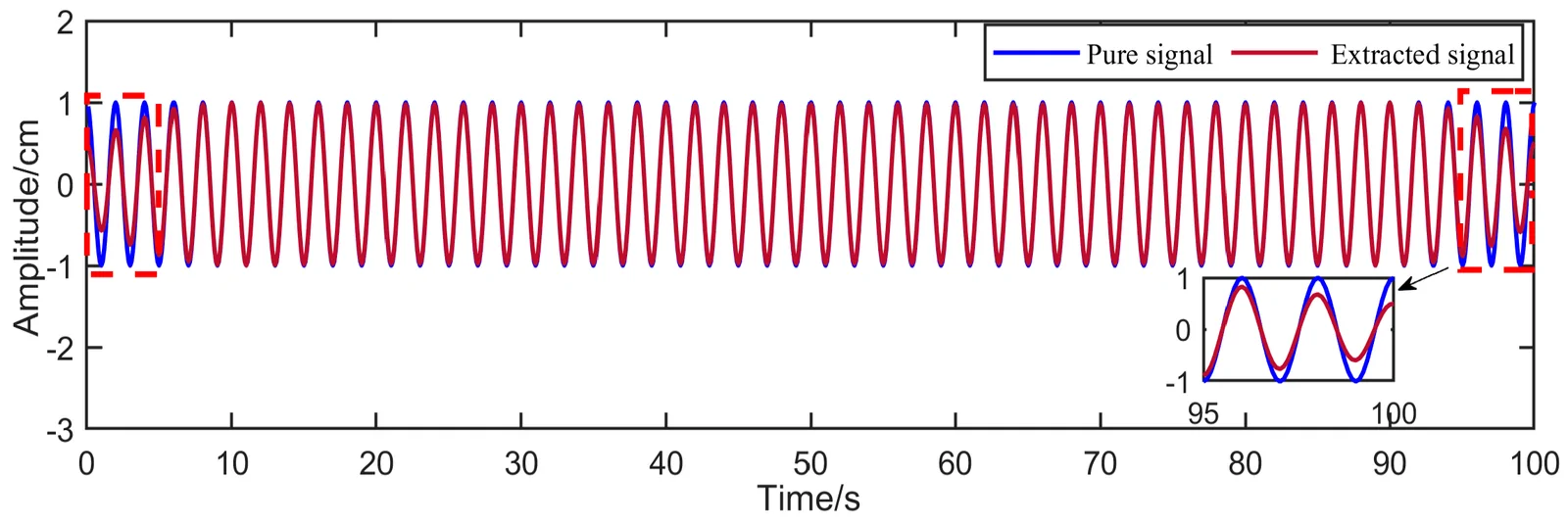
Illustration of the edge effects induced by the NTFT method on finite-length signal extraction.

A typical example of this phenomenon is shown in Figure 2, which uses a simulated signal at 0.5 Hz, with a sampling frequency of 10 Hz and a sampling duration of 100 s. The single-component signal is used to clearly demonstrate the boundary distortion without the influence of component interaction or mode mixing. As can be seen, although the extraction result remains generally stable in the central part of the signal, evident deviations occur between the extracted component and the reference component near the beginning and the end. These deviations are manifested as local amplitude distortion, waveform mismatch, and discontinuities in the boundary response. The results demonstrate that, even when the target frequency band has been reasonably constrained, conventional NTFT still cannot avoid the reduction in reliability of feature identification near the signal endpoints caused by edge effects. This limitation is particularly critical in structural dynamic response analysis when the signal of interest involves transient processes, short-duration variations, or local responses occurring near the endpoints. Under such circumstances, boundary distortion further restricts the engineering applicability of NTFT. Therefore, improving modal separation capability through band division alone is still insufficient for achieving high-accuracy extraction over the entire time domain, and a dedicated boundary-compensation strategy is required to mitigate the adverse influence of edge effects.

#### 2.2.3. Motivation for the MTM-eNTFT Method

The above analysis shows that NTFT faces two major limitations when directly applied to multimodal structural dynamic monitoring signals. First, in the absence of reliable prior frequency information, the target mode cannot be effectively separated through appropriate band division. Second, for finite-length signals, even when the target frequency band has been constrained, the edge effects inherent in conventional NTFT further reduce the extraction accuracy in the endpoint regions. The former issue can be attributed to insufficient prior constraints in the frequency domain, whereas the latter arises from inadequate boundary treatment in the time domain. Together, these two issues constrain the performance of NTFT in the analysis of complex multimodal signals.

To address the above limitations, this study proposes the MTM-eNTFT method, in which both the frequency-domain processing and the boundary treatment of NTFT are specifically improved. On the one hand, the multitaper method (MTM) is introduced to perform power spectral analysis of the original multimodal signal, thereby providing more robust and accurate prior modal frequency information. Based on this information, the NTFT processing bands corresponding to individual modes can be determined, which enhances the selectivity and reliability of target-component separation. On the other hand, an improved eNTFT framework is established through endpoint extension so as to improve the time-frequency representation and component extraction accuracy in the initial and terminal segments. By combining the prior-frequency identification capability of MTM with the boundary-compensation capability of eNTFT, the proposed method enables more accurate and more stable extraction of multimodal signal components.

### 2.3. Proposed MTM-eNTFT Method

To improve the accuracy of time-frequency feature extraction from multimodal structural dynamic monitoring signals, an improved NTFT method integrating multitaper spectral estimation and endpoint extension is proposed in this study, referred to as MTM-eNTFT. The proposed method consists of three main procedures. First, the MTM method is employed to estimate the power spectrum of the original response signal, from which robust frequency prior information is obtained for identifying the central frequencies and frequency bands of different modal components. Second, a preliminary band-limited NTFT analysis is performed within each modal frequency band, and the edge-effect length associated with each modal component is quantified. Third, a multiscale permutation entropy (MPE) constrained endpoint extension strategy is introduced to construct extended signal segments that are consistent with the original boundary segments in terms of dynamical complexity, dominant periodicity, and waveform similarity.

#### 2.3.1. MTM-Based Frequency Prior Identification and Modal Band Division

**Figure 3:**
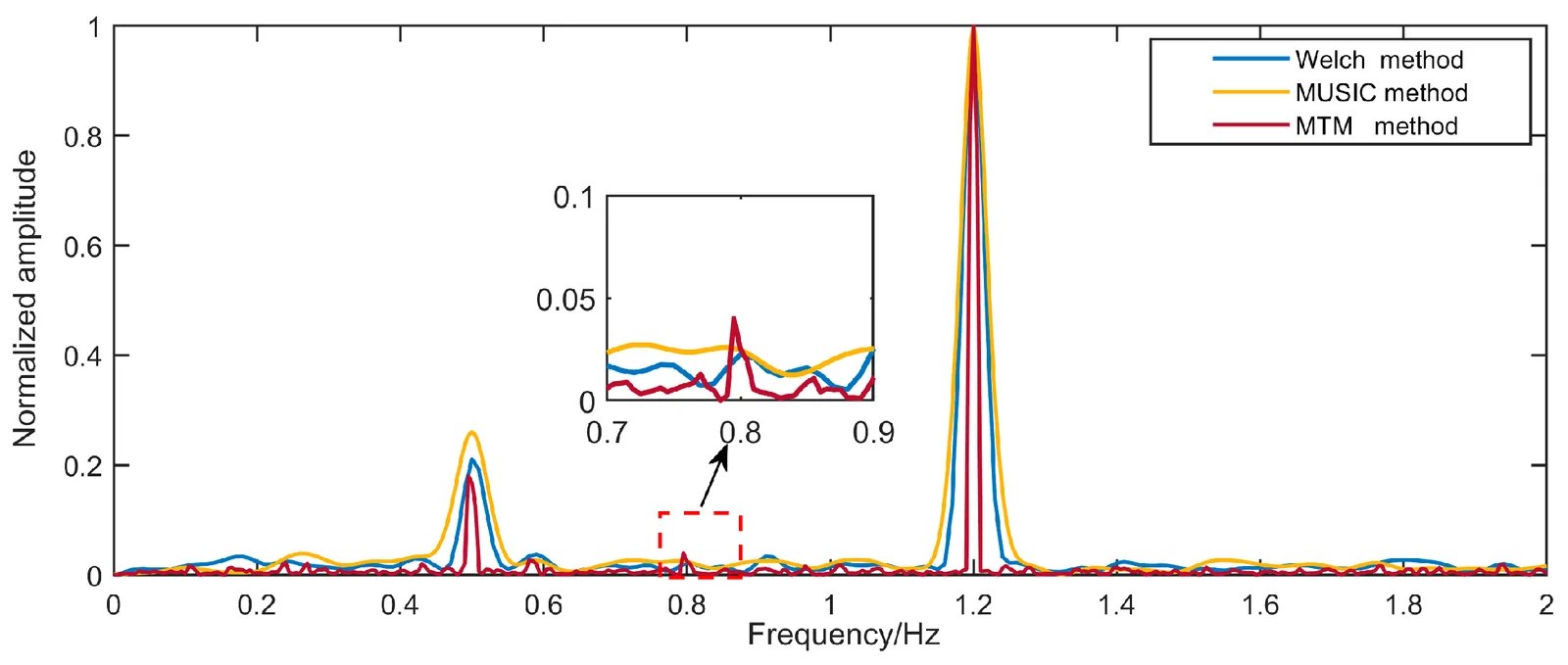
Normalized spectra of the simulated multimodal signal estimated using Welch, MUSIC, and MTM methods.

To determine a suitable spectral estimator for obtaining reliable frequency prior information, three commonly used power spectrum estimation methods, namely Windowed Averaged Periodogram Method (Welch), Multiple Signal Classification (MUSIC) and Multitaper Method (MTM), were compared using the simulated multimodal signal (identical to that in Figure 1). For reproducibility, Welch estimation was performed using a Hann window of 500 samples, an overlap of 100 samples (20%), and 1000 DFT points. For MUSIC, a Hann window of 256 samples with an overlap of 128 samples (50%) was used, and the parameter vector was set to [7, 1.1], where 7 denotes the maximum signal-subspace dimension and 1.1 is the eigenvalue-threshold factor used for noise subspace assignment. The MTM parameters, including the DPSS time-bandwidth product, number of tapers, and adaptive weighting scheme, are specified below Equation (6). For comparison, the resulting spectra were represented on matched frequency grids over the same frequency range, with a frequency-grid spacing of 0.01 Hz. None of the method-specific parameters was selected or adjusted using knowledge of the theoretical component frequencies. As shown in Figure 3, the spectrum estimated by the Welch method exhibits a relatively broad main lobe, which reduces its ability to distinguish closely spaced frequency components. Although MUSIC provides high spectral resolution, its performance is sensitive to the signal-to-noise ratio and the assumed model order, and it usually requires a higher computational cost. In contrast, MTM provides a more balanced performance in terms of spectral peak stability, noise robustness, and computational efficiency, making it more suitable for extracting frequency priors from finite-length structural monitoring signals. Therefore, MTM is adopted to provide frequency prior information for modal band division.

Let the discrete structural dynamic response signal be denoted by x(n),n=0,1,⋯,N−1, where N is the number of samples. To obtain reliable modal frequency prior information, MTM is adopted to estimate the power spectrum of the original signal. By applying a set of mutually orthogonal discrete prolate spheroidal sequences DPSS to the same data sequence, MTM can effectively reduce spectral leakage caused by finite-length signals and enhance the robustness of spectral peak identification under noisy conditions.

Let $v_{k}(n)$ denote the k−th DPSS taper and $X_{k}(f)$ be the corresponding eigenspectrum. The MTM power spectral estimate can be written as:

(6) $$\hat{P}MTM(f)=\sum {k=1}^{K}\bar{\mu }k(f){\left|X_{k}(f)\right|}^{2},\sum {k=1}^{K}{\bar{\mu }}_{k}(f)=1$$

where K is the number of tapers used for averaging, generally determined by the time-bandwidth product NW as K≤2NW−1, and ${\bar{\mu }}_{k}(f)$ is the normalized adaptive weight. In this study, NW is set to 1.5, and the corresponding number of tapers is selected as K=2. Compared with conventional periodogram-based or Welch spectral estimation methods, MTM provides better frequency resolution and noise robustness for finite-length structural response signals.

After obtaining ${\hat{P}}_{MTM}(f)$, the modal order and corresponding central frequencies are automatically identified from the dominant spectral peaks. Suppose that M dominant modes are identified, and their central frequencies are denoted by $f_{m},$ where m=1,2,…,M. The MTM-based modal frequency bands are determined using a fixed and reproducible peak-detection and boundary-search procedure. Before band selection, normalize the MTM power spectrum as $S_{N}(f)$. A spectral peak is accepted as a modal peak only when its normalized amplitude is higher than the peak-height threshold hp, its prominence is larger than the prominence threshold $p_{p}$, and its distance from adjacent accepted peaks is larger than the minimum peak distance. In this study, hp=0.05, $p_{p}$ = 0.03, and the minimum peak distance is set to three frequency bins. For each accepted modal peak ${\hat{f}}_{m}$, the left and right boundaries are searched outward from the peak. The first local valley whose normalized spectral amplitude is lower than the decay threshold $T_{m}=max[0.01,\;0.05S_{N}({\hat{f}}_{m})]$ is selected as the corresponding boundary. If no eligible local valley is found, the first frequency point at which the normalized spectrum remains below $T_{m}$ for three consecutive frequency bins is used as the energy-decay boundary. To avoid overlap between adjacent modal bands, the boundary search is limited by the midpoint between neighboring accepted modal peaks. Accordingly, all candidate spectral peaks and local valleys are detected algorithmically, while the accepted modal peaks, frequency-band boundaries, and resulting modal order are determined solely by the prescribed criteria. No peak or valley is manually accepted or rejected, and no frequency-band boundary or modal order is manually adjusted. The resulting modal frequency band is defined as:

(7) $$B_{m}=\left[f_{m}^{-},f_{m}^{+}\right]$$

where $f_{m}^{-}$ and $f_{m}^{+}$ are the automatically determined left and right boundaries, respectively. The subsequent NTFT analysis is performed only within the corresponding frequency band $B_{m}$. In this way, the original multimodal signal is transformed into several relatively independent narrow-band analysis tasks, which reduces modal aliasing and provides explicit frequency constraints for endpoint extension and modal reconstruction.

#### 2.3.2. MPE-Guided Endpoint Extension Strategy

After modal frequency-band division, a preliminary NTFT analysis is performed within the m−th frequency band $B_{m}$, and the corresponding initial modal component is denoted as ${\;x}_{m}(n)$ with length $N_{m}$. Since NTFT uses a local analysis window moving along the time axis, incomplete data support occurs when the window approaches the beginning or end of a finite-length signal, resulting in edge distortions in the extracted modal components. According to the definition of edge effects in Section 2.2, the edge-effect length of the m−th modal component can be expressed as:

(8) $$L_{e,m}=\left\lceil \frac{l_{m}}{\mu (\omega_{m})}\right\rceil$$

where $l_{m}$ is the half-width of the NTFT analysis window for the m−th mode, $\mu (\omega_{m})$ is the frequency-dependent scaling factor associated with the modal angular frequency $\omega_{m}$, and ⌈⋅⌉ denotes the ceiling operator. To effectively compensate for the edge-effect region, the endpoint extension length should not be smaller than $L_{e,m}$.

Although zero-padding, mirror extension, and simple periodic copying can increase the signal length, they may distort the dynamic characteristics of structural dynamic responses near the endpoints. To address this issue, MPE is introduced as a descriptor of local dynamical complexity and is combined with waveform correlation to adaptively select suitable endpoint extension segments.

For an arbitrary signal segment u, its MPE feature vector is defined as:

(9) $$H(u)={\left[h^{\left(1\right)}(u),h^{\left(2\right)}(u),\ldots ,h^{\left(S\right)}(u)\right]}^{T}$$

where S is the maximum scale factor, and $h^{\left(s\right)}(u)$ denotes the normalized permutation entropy of u at scale s, given by:

(10) $$h^{\left(s\right)}(u)=-\frac{1}{\ln(d!)}\sum_{r=1}^{d!}p_{r}^{\left(s\right)}\ln p_{r}^{\left(s\right)}$$

where d is the embedding dimension, and $p_{r}^{\left(s\right)}$ is the occurrence probability of the r−th ordinal pattern at scale s. The MPE feature vector characterizes the complexity variation of a signal segment over multiple temporal scales. It is used to measure the multiscale complexity consistency between the boundary segment and the internal candidate reference segment. It is not intended as an unconstrained criterion for length-selection [27,28,29].

**Figure 4:**
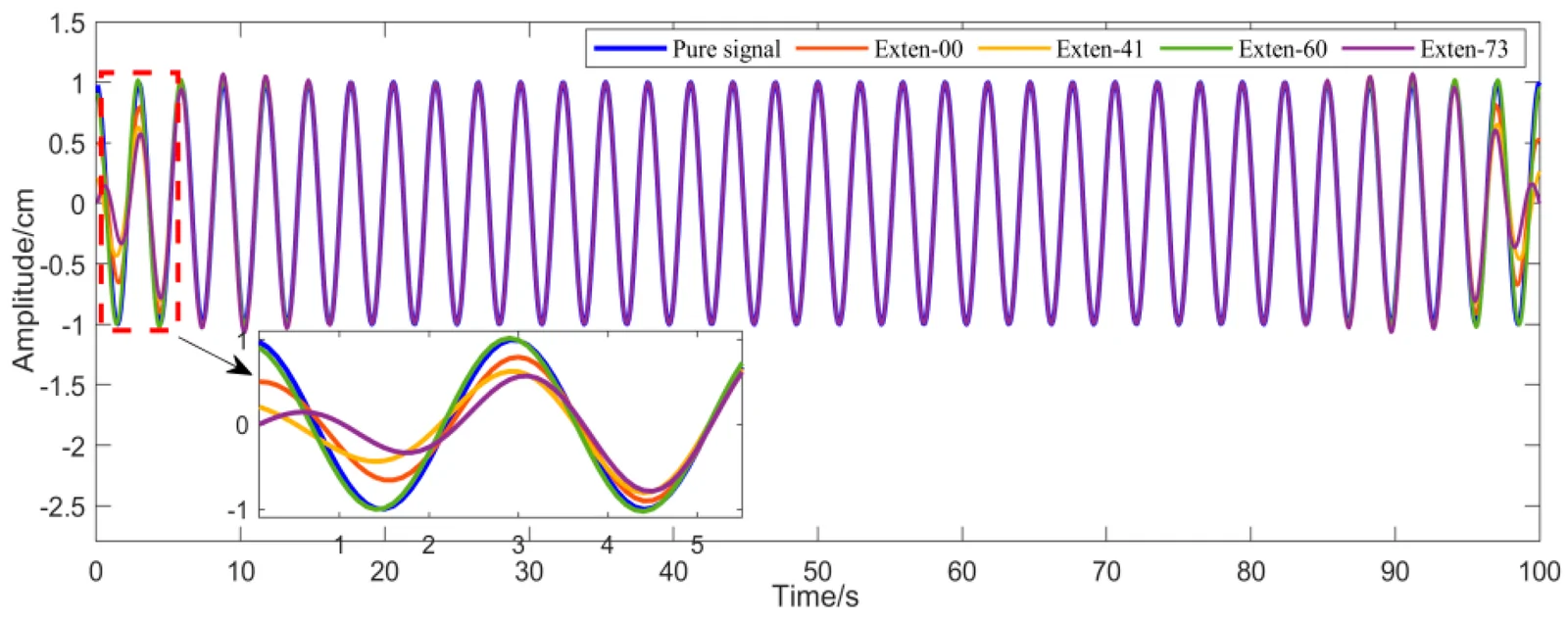
Comparison of signal extraction performance under different endpoint extension lengths.

To suppress the boundary distortion of band-limited NTFT, the endpoint extension length should be sufficiently long to cover the edge-effect region. Meanwhile, because each modal component is dominated by a narrow frequency band, the extension length should be consistent with the dominant modal period estimated by MTM, so as to reduce phase mismatch near the signal boundaries. Therefore, in the proposed MTM-eNTFT method, the extension length is not searched over all integer samples. Instead, a period-consistent admissible candidate set is first constructed by jointly considering the edge-effect length and the MTM-estimated dominant modal period. The final extension length is then selected by minimizing a joint MPE-guided criterion within this admissible set. For the m−th mode, the candidate extension length set is defined as:

(11) $$\Omega_{m}=\left\{L_{m,q}|L_{m,q}=round(q*\frac{f_{s}}{{\hat{f}}_{m}}),L_{e,m}\leq L_{m,q}\leq L_{m,max},q\in N^{+}\right\}$$

where ${\hat{f}}_{m}$ is the MTM-estimated dominant frequency of the m−th mode, and $L_{m,max}$ is the max search length allowed. This constraint ensures that the extension length covers the edge-effect region while being restricted to integer multiples of the MTM-estimated dominant modal period, thereby avoiding phase deviations caused by arbitrary endpoint extension. In Equation (11), since the dominant period is not necessarily an integer number of samples, $round(q*(f_{s}/{\hat{f}}_{m}))$ is used to generate implementable discrete candidate lengths. The lower bound $L_{e,m}$ ensures that the extension length covers the edge-effect region, while the upper bound $L_{m,max}$ prevents excessively long extensions. The necessity of imposing such constraints on the endpoint extension length is demonstrated in Figure 4 (the same simulated signal as in Figure 2). When the extension length is selected arbitrarily, the reconstructed component may still suffer from residual edge distortions or phase deviations, even though additional data have been padded at both endpoints. In particular, an excessively short extension cannot fully cover the edge-effect region, whereas an excessively long or non-period-consistent extension may introduce phase mismatch and unnecessary computational burden. Therefore, the extension length in the proposed MTM-eNTFT method is determined by jointly considering the edge-effect length and the dominant modal period obtained from MTM-based frequency prior information.

For any candidate length $L\in \Omega_{m}$, the boundary segment with length L is extracted from the left or right endpoint of $x_{m}(n)$ and denoted as $b_{m}^{\eta }(L)$, where η∈{L,R} indicates the left or right endpoint. Meanwhile, internal candidate reference segments with the same length are extracted from the non-edge region and denoted by $c_{m,j}(L)$, where j is the starting index. To avoid selecting segments affected by endpoint distortions, the admissible index set is denoted by $J_{m}(L)$. To jointly evaluate the local complexity consistency and waveform similarity between the boundary and candidate reference segments, a unified endpoint matching cost function is defined as:

(12) $$D_{m,j}^{\eta }(L)=\alpha {\left\|H\left(b_{m}^{\eta }(L)\right)-H\left(c_{m,j}(L)\right)\right\|}_{2}^{2}+(1-\alpha )\left[1-\rho \left(b_{m}^{\eta }(L),c_{m,j}(L)\right)\right]$$

where H(·) is the MPE feature vector, ρ(⋅,⋅) denotes the Pearson correlation coefficient, and α∈[0,1] is a weighting factor. A smaller value of $D_{m,j}^{\eta }(L)$ indicates that the internal reference segment is more consistent with the endpoint boundary segment in both multiscale complexity and waveform pattern. The weighting factor α controls the relative contribution of the MPE-consistency term and the waveform-correlation term. This cost function combines an entropy-based similarity term (via MPE) with a waveform-correlation term (via Pearson correlation), enabling the selection of reference segments that are similar to the boundary segments in both multiscale complexity and waveform pattern.

The optimal extension length of the m−th modal component is determined by minimizing the total matching cost of the two endpoints:

(13) $$L_{s,m}^{*}=\arg\underset{L\in \Omega_{m}}{min}\sum_{\eta \in \{L,R\}}\underset{j\in J_{m}(L)}{min}D_{m,j}^{\eta }(L)$$

Equation (13) indicates that the optimal endpoint-extension length is selected only from the admissible candidate set $\Omega_{m}$. Therefore, the selected length simultaneously satisfies the edge-effect coverage constraint and the dominant-period consistency constraint. The MPE-guided criterion is used only to rank the admissible candidates. Once $L_{s,m}^{*}$ is determined, the optimal reference segment index for each endpoint is obtained as:

(14) $$j_{m,\eta }^{*}=\arg\underset{j\in J_{m}(L_{s,m}^{*})}{min}D_{m,j}^{\eta }(L_{s,m}^{*}),\;\;\eta \in \{L,R\}$$

The extended modal signal ${\tilde{x}}_{m}$ is subsequently processed using band-limited NTFT, followed by inverse NTFT reconstruction. The central valid portion corresponding to the original signal duration is retained as the edge-compensated modal component ${\hat{x}}_{m}$:

(15) $${\hat{x}}_{m}(n)={\tilde{x}}_{m}(n+L_{s,m}^{*}),\;\;n=0,1,2,\ldots ,N-1$$

#### 2.3.3. Implementation Procedure and Flowchart of the MTM-eNTFT Method

**Figure 5:**
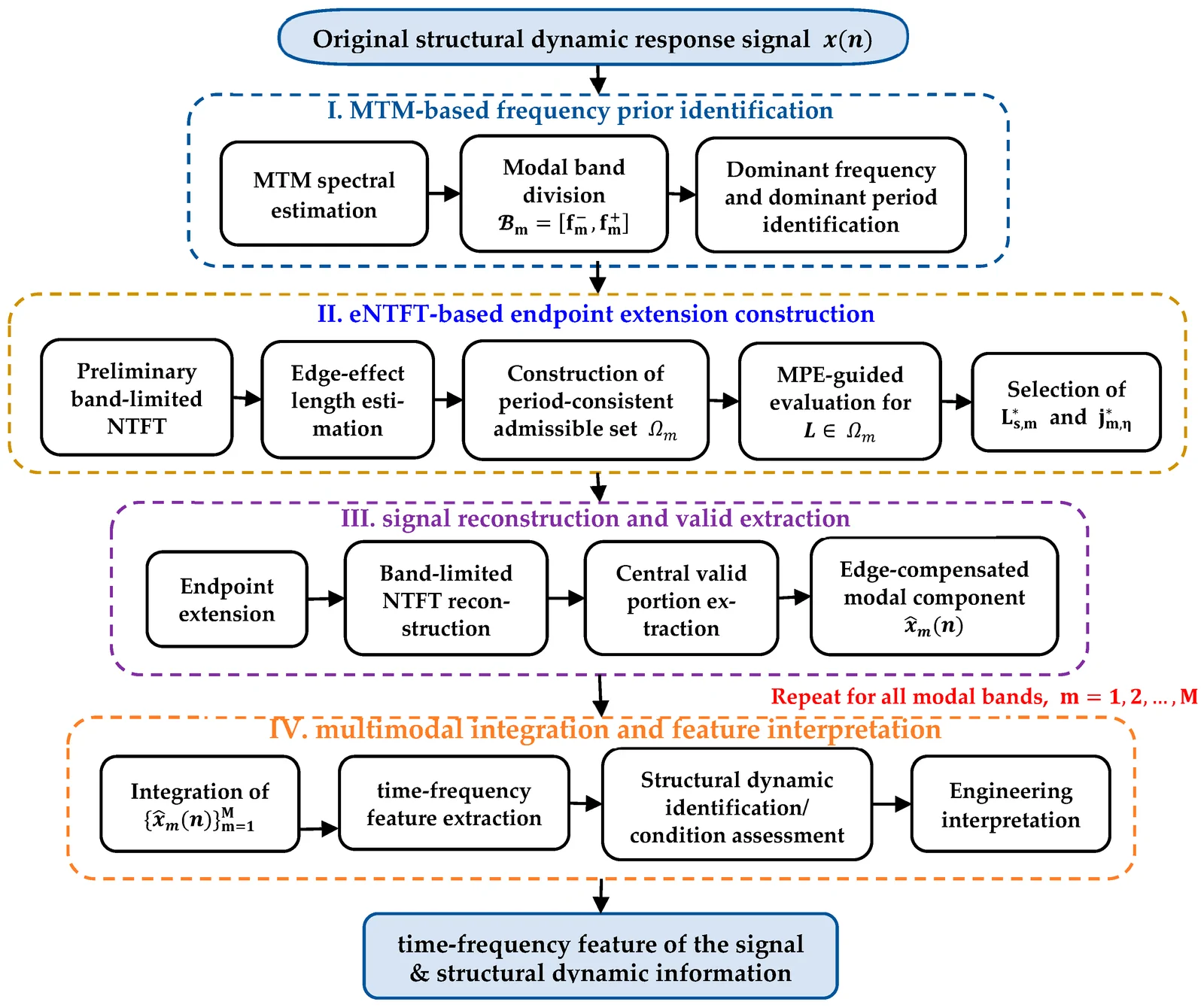
The flowchart of the MTM-eNTFT method.

The proposed MTM-eNTFT method can be summarized as a unified framework consisting of frequency prior identification, modal frequency-band division, edge-effect quantification, MPE-guided endpoint extension, and band-limited NTFT reconstruction. The workflow of the proposed MTM-eNTFT method is illustrated in Figure 5, and the implementation procedure is summarized into the following five steps.

Step 1: MTM power spectral estimation, modal-band division, and dominant-period identification. The power spectrum of the original structural dynamic response signal is estimated using MTM. The modal frequency bands $B_{m}=[f_{m}^{-},f_{m}^{+}]$ are determined. Meanwhile, the corresponding dominant modal period is given by ${\hat{P}}_{m}=f_{s}/{\hat{f}}_{m}$, which is then used to construct the period-consistent endpoint-extension candidate set.

Step 2: Preliminary band-limited NTFT analysis and edge-effect length estimation. Perform preliminary NTFT analysis within each modal frequency band $B_{m}$ to obtain the initial modal component $x_{m}(n)$. The corresponding edge-effect length $L_{e,m}$ is then estimated and used as the lower bound of the admissible endpoint-extension length.

Step 3: Period-consistent MPE-guided adaptive endpoint extension. For each initial modal component $x_{m}(n)$, the admissible candidate extension-length set $\Omega_{m}$ is first constructed according to the edge-effect length $L_{e,m}$ and the MTM-estimated dominant modal period ${\hat{P}}_{m}$. The optimal extension length $L_{s,m}^{*}$ is obtained by minimizing the joint MPE-guided criterion within $\Omega_{m}$, and the corresponding left- and right-end reference segments are then selected.

Step 4: Band-limited NTFT reconstruction of the endpoint-extended modal component. The endpoint-extended modal component $x_{m}(n)$ is processed using band-limited NTFT within the modal frequency band $B_{m}$. After inverse NTFT reconstruction, the central portion corresponding to the original signal duration is retained as the edge-compensated modal component ${\hat{x}}_{m}(n)$.

Step 5: Modal component output and time-frequency feature extraction. After the above procedure is completed for all modal frequency bands, the edge-compensated modal components ${\hat{x}}_{m}(n)$. Based on the reconstructed modal components, time-frequency features such as instantaneous frequency, instantaneous amplitude, energy distribution, and modal evolution characteristics can be further extracted for structural dynamic identification and condition assessment.

## 3. Simulation Experiment and Result Analysis

This section evaluates the component extraction performance of the proposed MTM-eNTFT method using a simulated multi-component signal. First, the simulated signal and its constituent components are introduced. The complete MTM-eNTFT procedure is then applied to determine the target frequency regions, optimize the endpoint extension, and reconstruct the three components. Subsequently, three groups of experiments are conducted: an ablation comparison with NTFT and MTM-NTFT, a comparison with VMD, SET, and CEEMDAN, and a robustness evaluation under different noise levels. Finally, the main findings and limitations of the proposed method are discussed.

### 3.1. Construction of the Simulated Signal

The simulated signal x(t) is composed of three theoretical signal components and additive Gaussian white noise:

(16) $$x(t)=x_{1}(t)+x_{2}(t)+x_{3}(t)+n(t),$$

where $x_{1}(t)$ is a low-frequency free-decay component used to simulate the decaying vibration response of a structure after an initial disturbance; $x_{2}(t)$ is a stationary harmonic component representing the stable vibration response under continuous environmental excitation; $x_{3}(t)$ is a local transient response component used to simulate finite-duration vibration caused by short-term local disturbance; and n(t) denotes Gaussian white noise. To reproduce a severe noise environment probably encountered in engineering monitoring, the signal-to-noise ratio of the noisy signal is set to −5 dB.

The sampling frequency is set to $f_{s}=10\;Hz$, and the total duration is 300 s. The three theoretical components are defined as follows:

(17) $$\left\{\begin{array}{c} x_{1}(t)=0.8e^{-t/700}\sin\left(2\pi \times 0.05t+\frac{2\pi }{3}\right),\;\;\;\;0\leq t\leq 300,\\ x_{2}(t)=0.15\sin\left(2\pi \times 0.30t\right),\;\;\;\;\;\;\;\;\;\;\;\;\;\;\;\;\;\;\;\;\;\;\;\;0\leq t\leq 300,\\ {\;\;\;\;x}_{3}(t)=0.5\sin\left(2\pi \times 0.15t\right)\sin\left[\frac{\pi (t-160)}{80}\right],\;\;160\leq t\leq 240,\\ x_{3}(t)=0,\;\;\;\;\;\;\;\;\;\;\;\;\;\;\;\;\;\;\;\;\;\;\;\;\;\;\;\;\;\;\;\;\;\;\;\;\;\;\;\;\;\;\;\;\;\;\;\;\;\;\;\;\;\;\;\;\;\;\;\;\;\;\;\;\;\;\;\mathrm{otherwise}. \end{array}\right.$$

**Figure 6:**
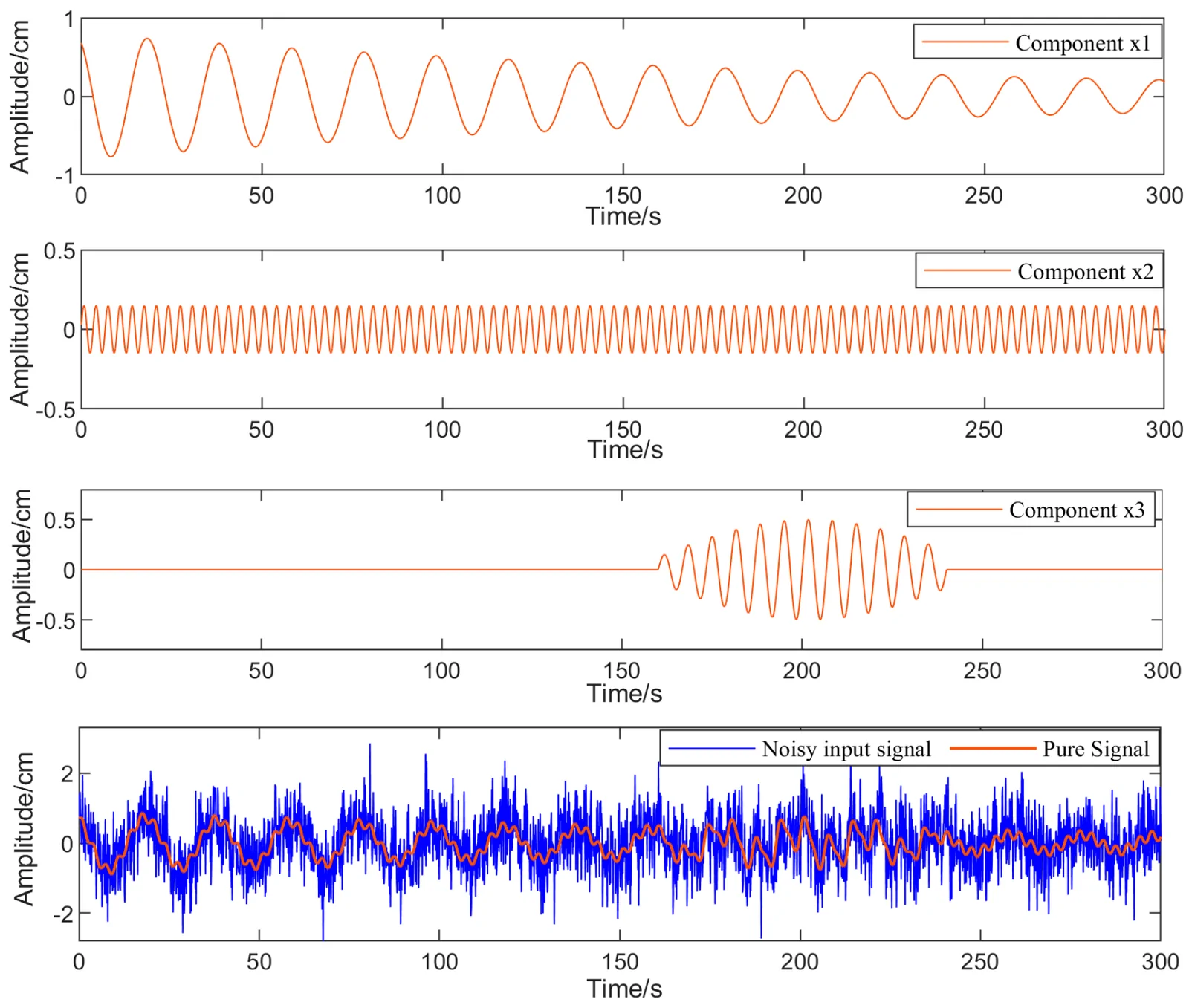
The noisy input signal and its theoretical components.

As shown in Equation (17), the dominant frequency of $x_{1}(t)$ is 0.05 Hz, corresponding to a low-frequency decaying vibration component. The dominant frequency of $x_{2}(t)$ is 0.30 Hz, corresponding to a continuous stationary vibration component. The dominant frequency of $x_{3}(t)$ is 0.15 Hz, and this component only exists within the time interval from 160 s to 240 s, reaching its maximum amplitude around t=200 s. Therefore, the simulated signal contains low-frequency decay, stationary vibration, and local non-stationary response characteristics, which makes it suitable for evaluating the applicability of different methods to complex multimodal noisy signals. The time-domain waveforms of the simulated signal and its theoretical components are shown in Figure 6.

### 3.2. Component Extraction Using the Proposed MTM-eNTFT Method

The proposed MTM-eNTFT method was first applied to the noisy input signal at an input SNR of −5 dB. The method consists of three main steps. First, the multitaper method (MTM) is used to identify the frequency regions associated with the target components. Second, multiscale permutation entropy (MPE) is employed to determine the endpoint extension length according to the criterion defined in Section 2. Finally, NTFT is applied to the extended signal, and the target components are reconstructed within the MTM-derived frequency regions. After reconstruction, the extended samples are removed so that the extracted components have the same duration as the original signal.

**Figure 7:**
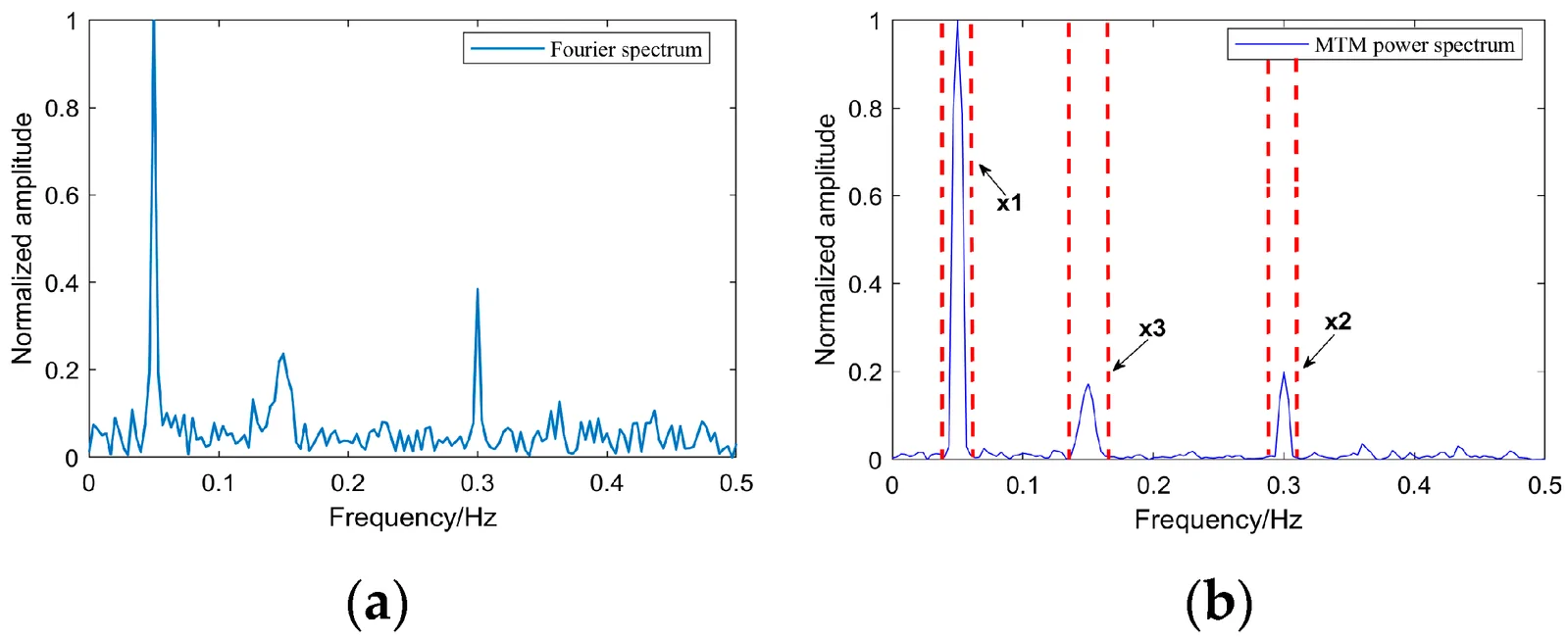
Spectral analysis results of the noisy input signal: (**a**) Fourier spectrum; (**b**) MTM power spectrum. The red dash line indsicate the frequency bands used to reconstruct the three target components.

Figure 7 compares the conventional Fourier spectrum (Figure 7a) with the MTM power spectrum (Figure 7b) of the noisy input signal. Although the input signal is severely contaminated by noise, the MTM spectrum provides distinguishable spectral peaks around the theoretical frequencies of the three components. Based on the peak locations and their neighboring spectral support, the frequency regions used for NTFT reconstruction were determined as [0.04∼0.06 Hz], [0.29∼0.31 Hz], and [0.13∼0.17 Hz], respectively. These regions were used consistently by MTM-NTFT and MTM-eNTFT in the subsequent experiments.

After the MTM-derived frequency regions were obtained, the dominant frequency $f_{m}$ of each component was identified from the corresponding MTM spectral peak. The candidate endpoint extension lengths were then constructed according to Equation (11). Specifically, each candidate length was constrained to be consistent with an integer multiple of the dominant period $T_{m}$ of the corresponding component. In addition, the candidate length had to be longer than the NTFT edge-effect interval $L_{e,m}=\left\lceil l_{win}/f_{m}\right\rceil$ and no longer than one third of the original signal length. In the present simulation, the NTFT half-window width $l_{win}$ was 15 samples. Since the original input signal length was N=3000 samples, the upper bound of the candidate length was N/3=1000 samples. The weighting factor employed in the endpoint matching cost function is set to 0.5. The MPE-based criterion (Equation (13)) described in Section 2 was then applied to this constrained candidate set, For each candidate set, MPE was calculated using an embedding dimension of m = 3, a time delay of τ = 1, and a scale range of s = 1:5. The length producing the minimum objective value was selected as the optimal endpoint extension length.

**Figure 8:**
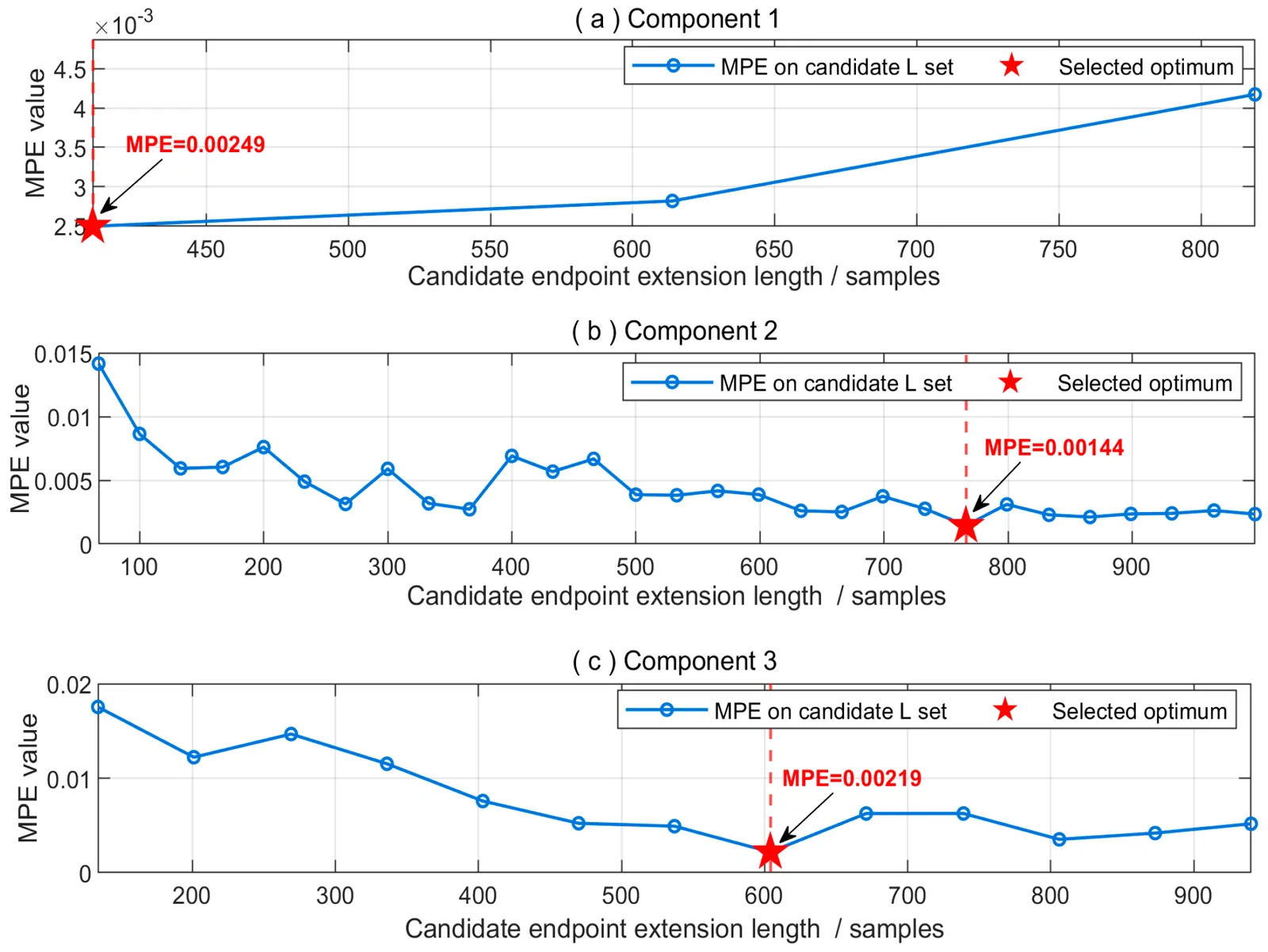
Relationship between MPE and endpoint extension length for each signal component in the MTM-eNTFT method.

As shown in Figure 8, the optimal extension lengths $L_{opt}$ obtained for the three components were 410, 766 and 604 samples, respectively. These values all belong to the candidate sets defined above. The corresponding candidate ranges and minimum objective values are summarized in Table 1.

**Figure 9:**
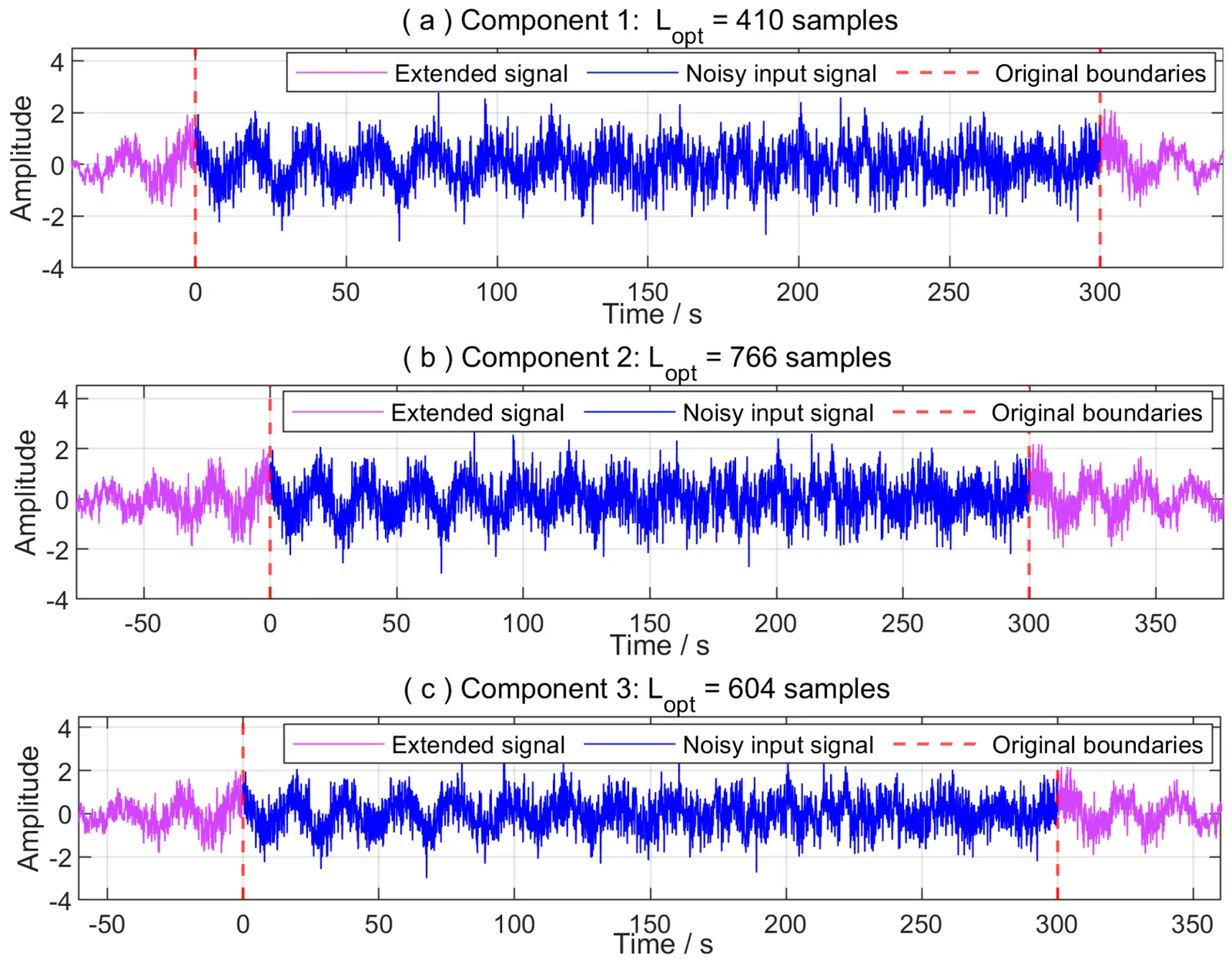
Endpoint extension results of each signal component.

The evident difference among the optimal extension lengths indicates that different signal components require different boundary data support, which confirms the necessity of component-adaptive endpoint extension rather than using a fixed extension length for all components. Based on the optimal extension lengths by Equation (13), the endpoint-extended signals of the three components are obtained, as shown in Figure 9.

Using the frequency regions and endpoint extension lengths reported in Table 1, the three components were reconstructed by NTFT and then truncated to the original time interval. The Time-Domain Fidelity (TDF) metric is introduced herein to quantify the waveform consistency between the extracted signal and its theoretical pure counterpart.

(18) $$TDF=\left(1-\frac{{\left\|\hat{s}(t)-s(t)\right\|}_{2}}{{\left\|s(t)\right\|}_{2}}\right)\times 100\%$$

where s denote the theoretical pure signal and $\hat{s}$ denote the extracted signal.

**Figure 10:**
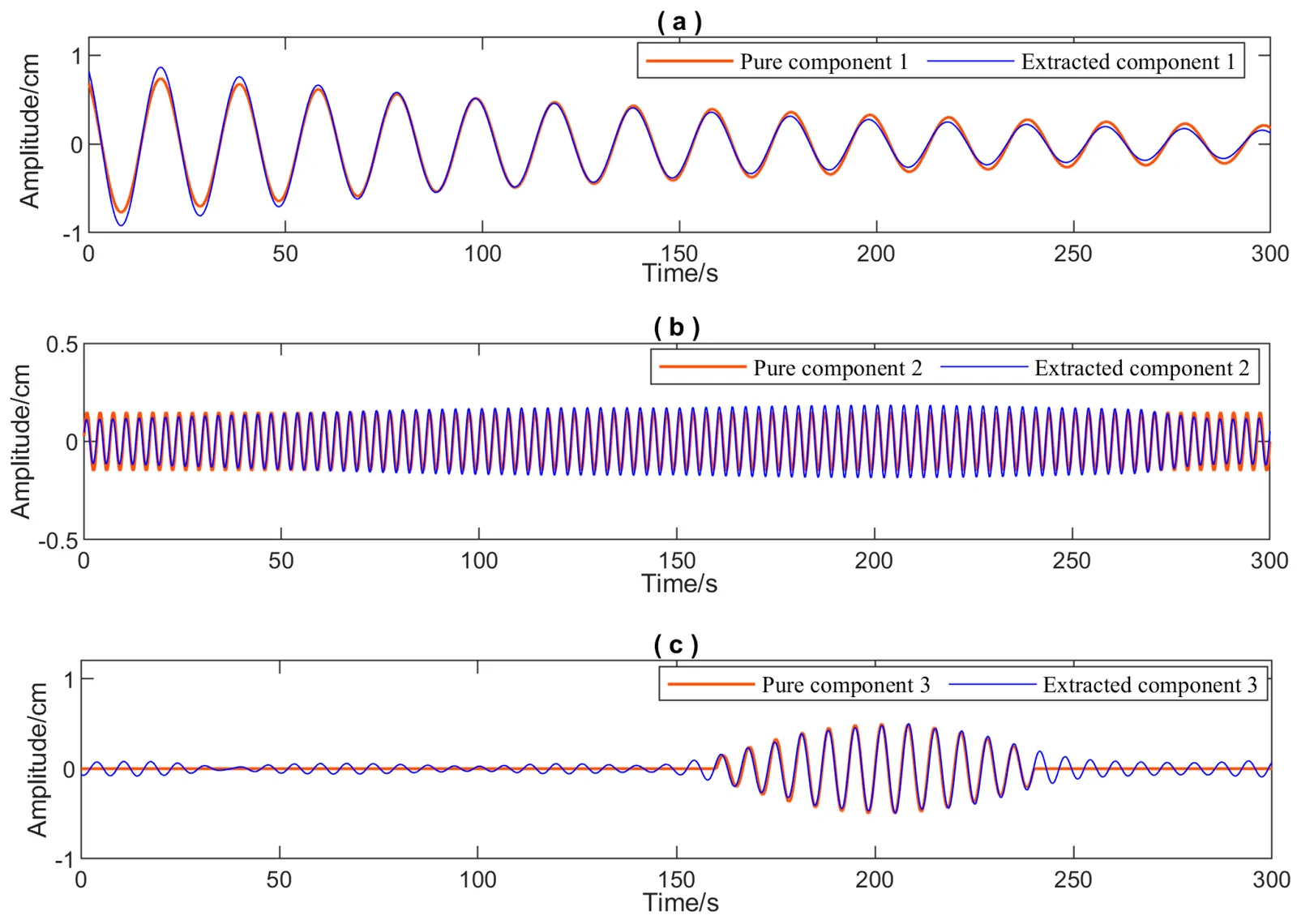
Components extracted by the proposed MTM-eNTFT method at an input SNR of −5 dB: (**a**) $x_{1}(t)$, (**b**) $x_{2}(t)$, and (**c**) $x_{3}(t)$.

Although the extracted signals exhibit slight time-domain waveform distortions, the filtered waveforms of the three components show good agreement with the theoretical pure signals, achieving a TDF of 80.86%. This demonstrates that MTM-eNTFT enables high-precision feature extraction in multimodal signal separation under strong noise conditions. Figure 10 compares the extracted components with their theoretical counterparts. The proposed method reproduces the decaying oscillation $x_{1}(t)$, the stationary harmonic component $x_{2}(t)$, and the localized transient component $x_{3}(t)$.

### 3.3. Comparative Experiments

To verify the extraction performance of the proposed method, comparative experiments were conducted to evaluate the effect of MTM-based frequency-band selection, the contribution of MPE-based endpoint extension, and the performance of the proposed method relative to several commonly used decomposition methods. The comparative experiments were designed in three parts. In the first part, the proposed method was compared with the conventional NTFT and the MTM-NTFT without endpoint extension, aiming to demonstrate the necessity of introducing the endpoint-extension strategy. In the second part, MTM-eNTFT was evaluated against several representative signal analysis methods, namely VMD, SET, and CEEMDAN, to verify its effectiveness. In the third part, the robustness of MTM-eNTFT was examined under two different noise conditions (SNR = −5 dB and SNR = −10 dB). To objectively quantify the reliability and effectiveness of MTM-eNTFT, three accuracy metrics were further adopted: the root mean square error (RMSE), the Pearson correlation coefficient (PCC), and the signal-to-noise ratio (SNR).

(19) $$RMSE=\sqrt{\frac{1}{N}\sum_{i=1}^{N}{\left(s_{i}-{\hat{s}}_{i}\right)}^{2}}$$

(20) $$PCC=\frac{\sum_{i=1}^{N}\left(s_{i}-\bar{s}\right)\left({\hat{s}}_{i}-\bar{\hat{s}}\right)}{\sqrt{\sum_{i=1}^{N}{\left(s_{i}-\bar{s}\right)}^{2}}\cdot \sqrt{\sum_{i=1}^{N}{\left({\hat{s}}_{i}-\bar{\hat{s}}\right)}^{2}}}$$

(21) $$SNR=10\log_{10}\frac{\sum_{i=1}^{N}s_{i}^{2}}{\sum_{i=1}^{N}{\left(s_{i}-{\hat{s}}_{i}\right)}^{2}}$$

where s denote the theoretical pure signal and $\hat{s}$ denote the extracted signal.

#### 3.3.1. Comparison of NTFT, MTM-NTFT, and MTM-eNTFT

To clarify the respective contributions of MTM-based frequency-band selection and MPE-based endpoint extension, three NTFT-based configurations were compared over 30 independent noise trials at an input SNR of −5 dB. The first configuration, denoted as NTFT, applied the conventional NTFT directly to the finite-length noisy signal. The second configuration, denoted as MTM-NTFT, used the frequency regions determined by MTM but did not apply endpoint extension. The third configuration, denoted as MTM-eNTFT, used both the MTM-derived frequency regions and the MPE-based endpoint extension. The NTFT parameters, including window type (Gaussian window), window length ($L_{win}=15$), time step (Δt=0.1 s), and frequency step (Δf=0.01 Hz), were kept identical in the three configurations.

In this design, the difference between NTFT and MTM-NTFT reflects the contribution of MTM-based frequency-region determination, whereas the difference between MTM-NTFT and MTM-eNTFT reflects the contribution of endpoint extension. Since endpoint distortion mainly occurs within the edge-effect regions, an additional edge-region RMSE was calculated to evaluate the reconstruction accuracy near the signal boundaries. According to the statistical results in Table 1, the longest edge-effect interval length is 308 samples. To fully cover the region affected by the edge effect, data from 500 samples at each end of the signal are selected for calculating the edge RMSE. The edge RMSE was calculated as:

(22) $${RMSE}_{e}=\sqrt{\frac{1}{2L_{e}}\sum_{i\in \Omega_{e}}{\left[x(i)-\hat{x}(i)\right]}^{2}}$$

where $\Omega_{e}=\left\{1,\cdots ,L_{e}\right\}\cup \left\{N-L_{e}+1,\ldots ,N\right\}$. RMSE, PCC, output SNR, TDF, and edge RMSE were calculated separately for each trial. The results in Table 2 are reported as the mean ± standard deviation over the 30 independent trials, with the values in brackets denoting the two-sided 95% confidence intervals of the mean calculated using Student’s t−distribution.

**Figure 11:**
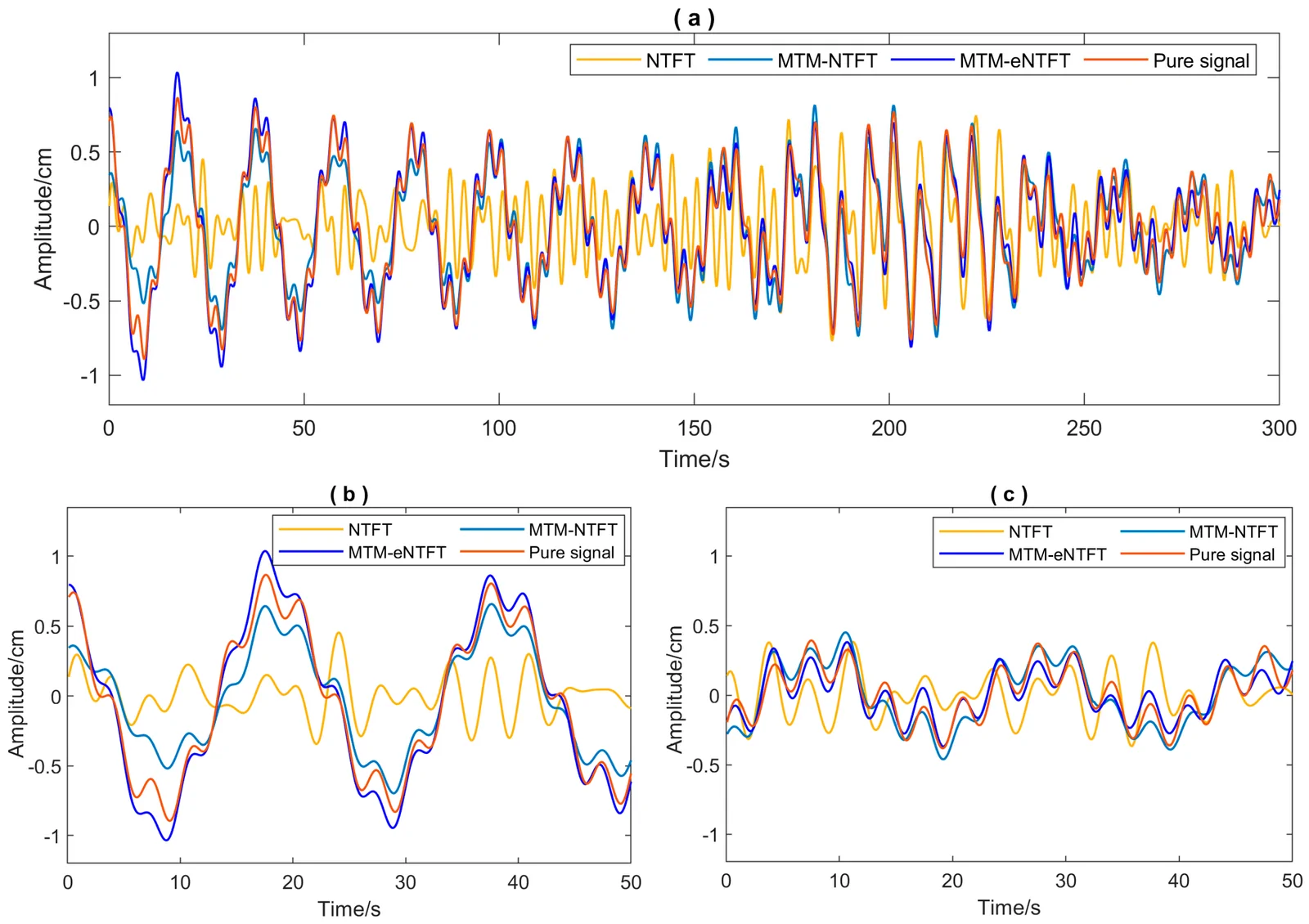
Ablation comparison of NTFT, MTM-NTFT, and MTM-eNTFT: (**a**) complete reconstructed signal; (**b**) enlarged left-boundary region; (**c**) enlarged right-boundary region.

Figure 11 compares the signal extraction results of the three methods for a single realization generated using a fixed random seed. As shown in Figure 11a, for the heavily noise-contaminated multimodal signals, direct application of the NTFT method without prior frequency band segmentation fails to extract effective signals—a conclusion corroborated by the quantitative results in Table 2. Figure 11b,c illustrate the extraction results at the left and right endpoints of the signals, respectively. Compared with the MTM-NTFT method, the waveforms extracted by MTM-eNTFT show higher consistency with the theoretical pure signals, indicating that the proposed endpoint extension strategy is reliable for processing such signals.

The quantitative results over the 30 independent noise trials are listed in Table 2. Conventional NTFT achieved a mean RMSE of 0.3578 ± 0.0465, but its mean PCC remained low at 0.3830 ± 0.0390, indicating that conventional NTFT alone was insufficient under the −5 dB noise condition. After MTM-based frequency-band selection was introduced, MTM-NTFT substantially improved the reconstruction performance, achieving an RMSE of 0.1085 ± 0.0128, a PCC of 0.9582 ± 0.0048, an output SNR of 10.7957 ± 1.0241 dB, and a TDF of 71.15 ± 3.40%. Further incorporating MPE-based endpoint extension, MTM-eNTFT achieved an RMSE of 0.0716 ± 0.0045, a PCC of 0.9833 ± 0.0020, an output SNR of 14.4109 ± 0.5400 dB, and a TDF of 80.96 ± 1.20%. Notably, the mean edge RMSE decreased from 0.1415 ± 0.0190 for MTM-NTFT to 0.0850 ± 0.0115 for MTM-eNTFT, corresponding to a reduction of approximately 39.9%. These repeated-trial results confirm that MPE-based endpoint extension consistently alleviates boundary effects and improves reconstruction accuracy under different noise realizations.

#### 3.3.2. Comparison with VMD, SET, and CEEMDAN

The proposed MTM-eNTFT method was further compared with VMD, SET, and CEEMDAN to evaluate its effectiveness in extracting noisy multimodal signals. All methods were evaluated over the same 30 independent noise trials at an input SNR of −5 dB. Within each trial, CEEMDAN, VMD, SET, and MTM-eNTFT were applied to the same noisy realization to ensure a paired and fair comparison. The clean signal x(t), defined as the sum of the theoretical target components, was used as the reference. For each method, the reconstructed signal $\;\hat{x}\;(t)$ was obtained by summing the selected modes, IMFs, or reconstructed frequency-band components. For each method, RMSE, PCC, output SNR, and TDF were calculated separately over the full signal length in every trial and were subsequently summarized over the 30 independent trials. Thus, this comparison evaluates the overall reconstruction performance rather than the extraction accuracy of a specific local component.

In each trial, the target frequency bands were identified from the noisy input and used consistently for all methods. Parameter tuning and component selection were conducted without access to the clean reference. Let $Y_{r}\left[k\right]$ and ${\hat{X}}_{m,r,\theta }\left[k\right]$ denote the discrete Fourier transforms of the noisy input and the reconstruction produced by method m with candidate parameter setting θ in trial r, respectively. Let $B_{r}$ denote the union of the identified target-frequency bins and ${\bar{B}}_{r}$ its complement over the nonnegative-frequency grid. The common reference-free criterion was defined as:

(23) $$J_{m,r}\left(\theta \right)=\frac{\sum_{k\in B_{r}}{\left|Y_{r}\left[k\right]-{\hat{X}}_{m,r,\theta }\left[k\right]\right|}^{2}}{2\sum_{k\in B_{r}}{\left|Y_{r}\left[k\right]\right|}^{2}+{ℇ}_{r}}+\frac{\sum_{k\in {\bar{B}}_{r}}{\left|{\hat{X}}_{m,r,\theta }\left[k\right]\right|}^{2}}{2\sum_{k\in {\bar{B}}_{r}}{\left|Y_{r}\left[k\right]\right|}^{2}+{ℇ}_{r}}$$

where $\varepsilon_{r}=10^{-12}\sum_{k}{\left|Y_{r}\left[k\right]\right|}^{2}$ prevents division by zero. The two terms quantify the normalized reconstruction mismatch within the target bands and the normalized residual energy outside those bands, respectively. Region-wise normalization renders both terms dimensionless, and equal weights were assigned to avoid favoring either objective. The criterion does not involve the clean reference. The VMD and SET parameters were selected once and subsequently fixed for all 30 trials rather than being reselected for each noise realization. Specifically, all candidate combinations were evaluated on the same 30 noisy realizations, and the setting minimizing the mean criterion was retained $\theta_{m}^{*}=\begin{array}{c} argmin\\ \theta \in \Theta_{m} \end{array}\frac{1}{30}\sum_{r=1}^{30}J_{m,r}\left(\theta \right)$. For VMD, the search covered all combinations of α∈500,1000,2000,3000,5000 and $\tau \in \left\{0,10^{-4}\right\}$, with K=3 and a convergence tolerance of $10^{-7}$; the selected setting was $\left(\alpha ,\tau )=(5000,10^{-4}\right)$. For SET, the periodic Hann-window length and IF tolerance were searched over $L_{Hann}\in \{128,192,256\}$ samples and $\delta_{IF}\in \{0.75,1.0,1.25\}$, respectively, yielding ($L_{Hann}$,$\delta_{IF}$) = (256,1.25). The remaining SET settings were $N_{FFT}=2048$, a hop size of 4 samples, and a frequency-grid spacing of 0.01 Hz. Each selected parameter combination was applied to all 30 trials; therefore, per-realization selection frequencies are not applicable. CEEMDAN used 300 ensemble realizations, an added-noise amplitude of 0.12, a maximum of 150 sifting iterations, and a stopping criterion of 0.08; these parameters were fixed a priori. For VMD and CEEMDAN, the mode or IMF with its dominant frequency inside each target band and the highest normalized in-band energy ratio was selected. This procedure used neither the clean reference nor reference-based gain correction. The MTM-eNTFT settings were identical to those used in Section 3.2. The clean reference was introduced only after parameter and component selection to compute the evaluation metrics reported in Table 3.

**Figure 12:**
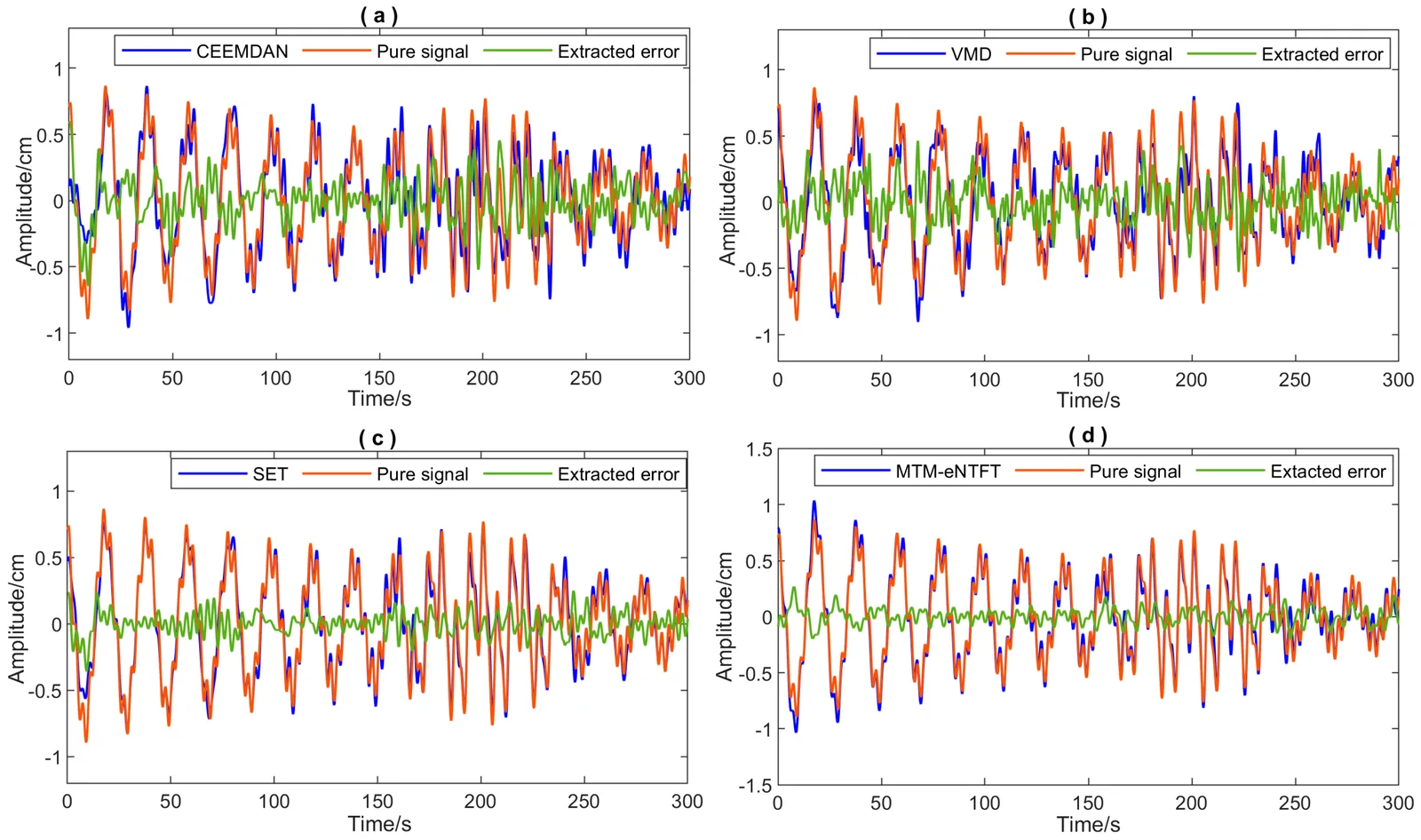
Reconstructed signals obtained by VMD, SET, CEEMDAN, and MTM-eNTFT: (**a**) CEEMDAN reconstructed signal; (**b**) VMD reconstructed signal; (**c**) SET reconstructed signal; (**d**) MTM-eNTFT reconstructed signal.

Figure 12 compares the reconstructed signals obtained using CEEMDAN, VMD, SET, and the proposed MTM-eNTFT. CEEMDAN and VMD can extract the main waveform component, but visible residual fluctuations and local amplitude deviations remain in the reconstructed signals, reflecting their limited robustness under strong noise. SET produces a smoother and more consistent reconstruction, indicating improved time-frequency concentration. However, the MTM-eNTFT result shows the best agreement with the reference signal in terms of waveform morphology, phase consistency, and amplitude preservation. This visual comparison suggests that the proposed method is more effective in suppressing noise while retaining the nonstationary characteristics of the target signal. Figure 12 shows one representative realization for visual illustration. The quantitative comparison reported in Table 3 is based on all 30 independent noise trials.

The quantitative results over the 30 independent noise trials are summarized in Table 3. Compared with CEEMDAN, VMD, and SET, MTM-eNTFT achieved the lowest mean RMSE of 0.0716 ± 0.0045 and the highest mean PCC, output SNR, and TDF values of 0.9833 ± 0.0020, 14.4109 ± 0.5400 dB, and 80.96 ± 1.20%, respectively. Among the three competing methods, SET exhibited the best overall performance, with an RMSE of 0.0830 ± 0.0055, a PCC of 0.9755 ± 0.0030, an output SNR of 13.1265 ± 0.5758 dB, and a TDF of 77.88 ± 1.46%. Relative to SET, MTM-eNTFT reduced the mean RMSE by approximately 13.7% and increased the mean output SNR by approximately 1.28 dB. These repeated-trial results demonstrate that the performance advantage of MTM-eNTFT at −5 dB is reproducible across independently generated noise realizations rather than being determined by a single noise sequence.

#### 3.3.3. Comparison Between −5 dB and −10 dB Noise Conditions

To further illustrate the behavior of the proposed method under more challenging noise, an additional single-realization simulation at an input SNR of −10 dB was carried out. The clean signal was contaminated by additive white Gaussian noise using the same procedure as in the −5 dB case, while all other simulation settings remained unchanged. MTM-eNTFT was then applied to the noisy signal, following the complete procedure including MTM-based frequency-region identification, MPE-based endpoint extension, and NTFT-based component reconstruction. The main algorithmic parameters were kept consistent with those in the −5 dB experiment. The identified frequency regions and the optimized extension lengths were, however, adaptively recalculated from the noisy signal.

Since the quantitative results for the −5 dB case have been reported in Section 3.3.1 and Section 3.3.2, they are not repeated here. For the newly added −10 dB case, MTM-eNTFT achieves an RMSE of 0.3594, a PCC of 0.6459, an output SNR of 0.3941 dB, and a TDF of 4.44%. Compared with the −5 dB results, the reconstruction performance deteriorates substantially at −10 dB because of the stronger noise masking. The output SNR increases from the input level of −10 dB to 0.3941 dB, indicating aggregate noise attenuation. However, the RMSE of 0.3594 and PCC of 0.6459 also indicate considerable residual errors. It should be emphasized that these metrics are calculated for the summed reconstructed signal and therefore characterize the overall reconstruction rather than the faithful recovery of each individual component. A component-wise evaluation was further conducted to distinguish aggregate denoising from individual-component recovery. At −10 dB, the RMSE and PCC are 0.1578 and 0.8811 for Component 1, 0.2538 and 0.3661 for Component 2, and 0.1752 and 0.6447 for Component 3, respectively. These results indicate that Component 1 remains comparatively recoverable, whereas Component 3 is only partially recovered and Component 2 is most strongly affected by the severe noise.

**Figure 13:**
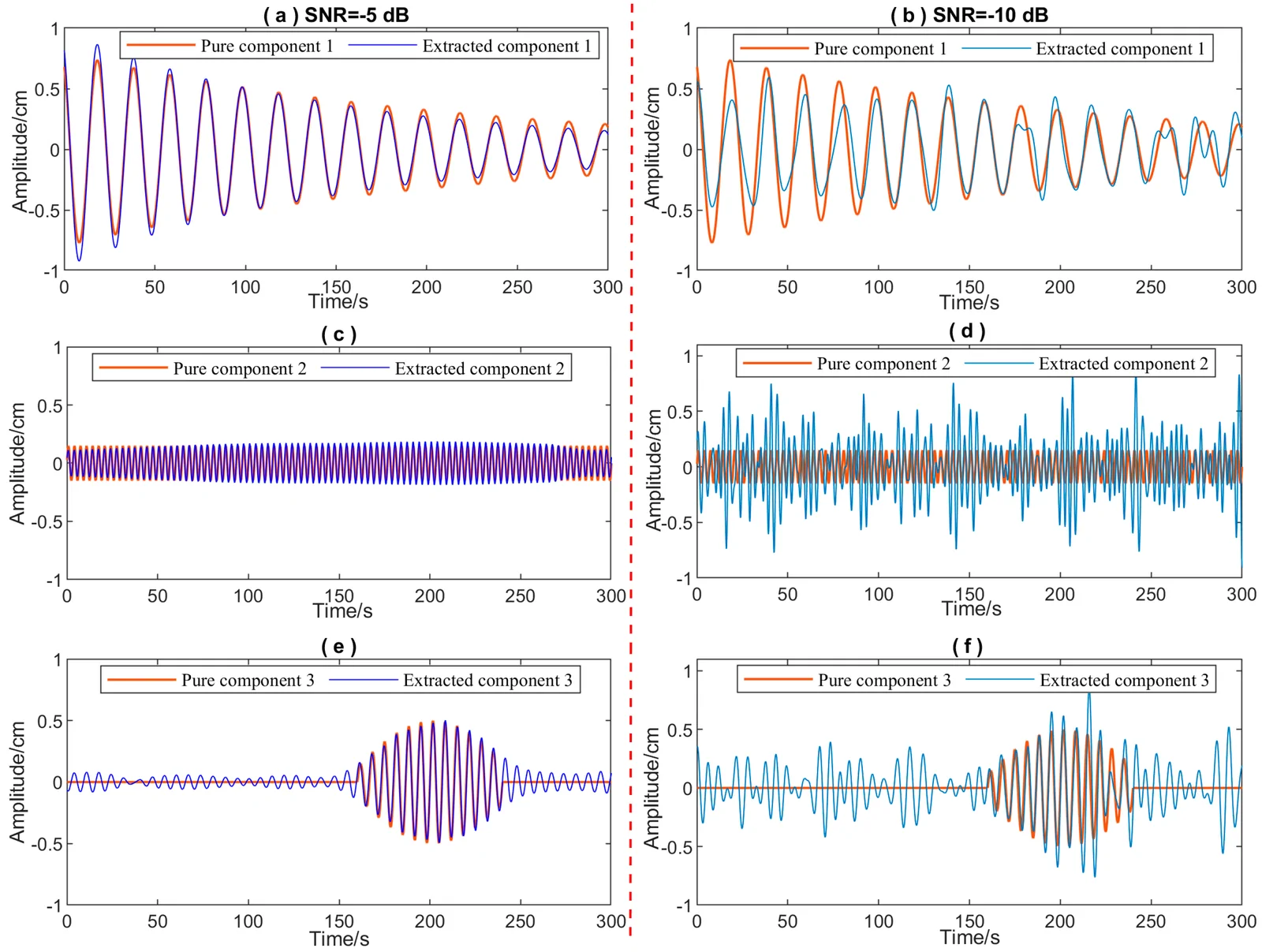
Time-domain comparison of the three components extracted by MTM-eNTFT under different input SNR conditions: (**a**,**c**,**e**) extracted Components 1–3 at −5 dB; (**b**,**d**,**f**) extracted Components 1–3 at −10 dB. The first column corresponds to the −5 dB case, and the second column corresponds to the −10 dB case.

**Figure 14:**
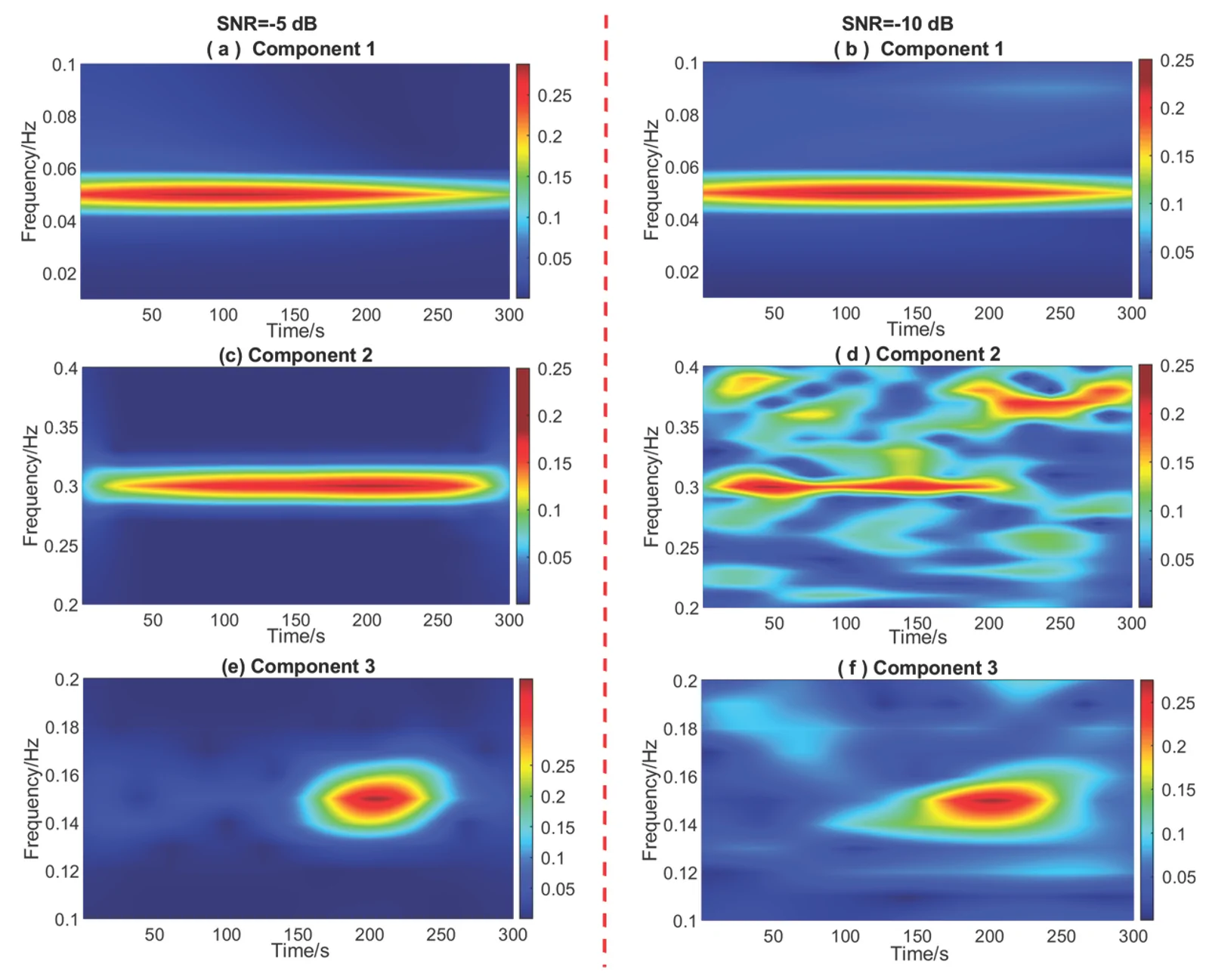
Time-frequency representations of the three components extracted by MTM-eNTFT under different input SNR conditions: (**a**,**c**,**e**) extracted Components 1–3 at −5 dB; (**b**,**d**,**f**) extracted Components 1–3 at −10 dB.

Figure 13 compares the time-domain extraction results of the three components under the −5 dB and −10 dB input SNR conditions, and Figure 14 presents the corresponding time-frequency representations. Compared with the −5 dB case, the extracted components at −10 dB exhibit more pronounced local fluctuations and amplitude deviations, while their time-frequency representations show increased background energy and reduced energy concentration. The degradation is component dependent: Component 1 retains the clearest principal structure, Component 3 is only partially recovered, and Component 2 is most strongly affected by noise. Therefore, the remaining visual correspondence should not be interpreted as accurate reconstruction of all individual components.

Overall, the −10 dB results demonstrate aggregate noise attenuation and partial preservation of some dominant temporal and spectral characteristics. However, they do not establish faithful modal-component reconstruction under such severe noise. It should be emphasized that all aggregate and component-wise metrics reported at −10 dB were obtained from this single noise realization. Therefore, these values are illustrative only and do not establish statistical stability or generalizable performance at −10 dB.

### 3.4. Discussion

The comparative results in Section 3.3 demonstrate that the performance improvement of MTM-eNTFT arises from the complementary effects of robust frequency-region identification and endpoint compensation. MTM provides stable frequency prior information for suppressing out-of-band noise, whereas the endpoint-extension procedure reduces the boundary distortion caused by finite-length truncation. In this procedure, MPE plays an important role in characterizing the multiscale dynamic complexity of the endpoint and candidate reference segments. The MPE-guided criterion ranks the candidates that satisfy the edge-effect coverage and dominant-period consistency constraints. Therefore, the extension process combines entropy-based similarity with modal periodicity rather than relying on unconstrained waveform matching. This mechanism accounts for the simultaneous improvement in global reconstruction accuracy and boundary reliability.

From a methodological perspective, MTM-eNTFT is a targeted extension of conventional NTFT rather than a new time-frequency transform. Its main contribution lies in establishing a frequency-prior-guided and entropy-constrained reconstruction framework for noisy finite-length signals. The ablation results indicate that MTM-based frequency-region selection primarily contributes to global noise suppression, while MPE-based endpoint extension provides additional improvement near the signal boundaries. Their integration therefore enables more reliable component reconstruction than either procedure alone. Comparisons with CEEMDAN, VMD, and SET confirm that explicit frequency priors and boundary information are advantageous when the target modes occupy distinguishable spectral regions. Under the −10 dB condition, however, severe noise contamination strongly masks the low-amplitude and localized features of the components, leaving less effective signal information available for reconstruction. The resulting performance degradation therefore reflects the reduced applicability of the proposed framework under extremely low-SNR conditions, rather than the effect of any single processing step.

The conclusions should be interpreted within the validation conditions considered in this study. MTM-eNTFT is most suitable for finite-length signals whose target components retain identifiable temporal and spectral features; its effectiveness may decrease when these features are substantially obscured by extreme noise or when closely spaced components exhibit strong spectral overlap. The present results establish the feasibility and comparative effectiveness of the proposed framework under representative conditions, while its broader robustness and computational scalability require further investigation.

## 4. A Case Study

The dynamic response of super-tall buildings under wind excitation is an important issue in structural health monitoring. Identification of dominant frequency components and their time-varying energy distributions is useful for characterizing measured structural responses under complex wind environments. In this section, a field acceleration record collected from a super-tall building in Hong Kong during a high-wind event is used as an application example. The proposed MTM-eNTFT method is applied to examine its capability for dominant frequency component extraction and time-frequency characterization under practical noise conditions.

### 4.1. Monitoring Configuration and Data Description

The CLP Wind Tunnel Laboratory of the Hong Kong University of Science and Technology conducted long-term monitoring of the wind-induced dynamic response of a super-tall building in Hong Kong. The building has an approximately rectangular plan, with a length-to-width ratio of about 4:1. It consists of 68 stories and has a total height of approximately 260 m. The X-direction of the building is oriented approximately 42° west of north. Owing to the relatively open surrounding environment at the roof level, an automatic structural dynamic monitoring system was installed on the roof to measure the wind-induced responses of the building.

**Figure 15:**
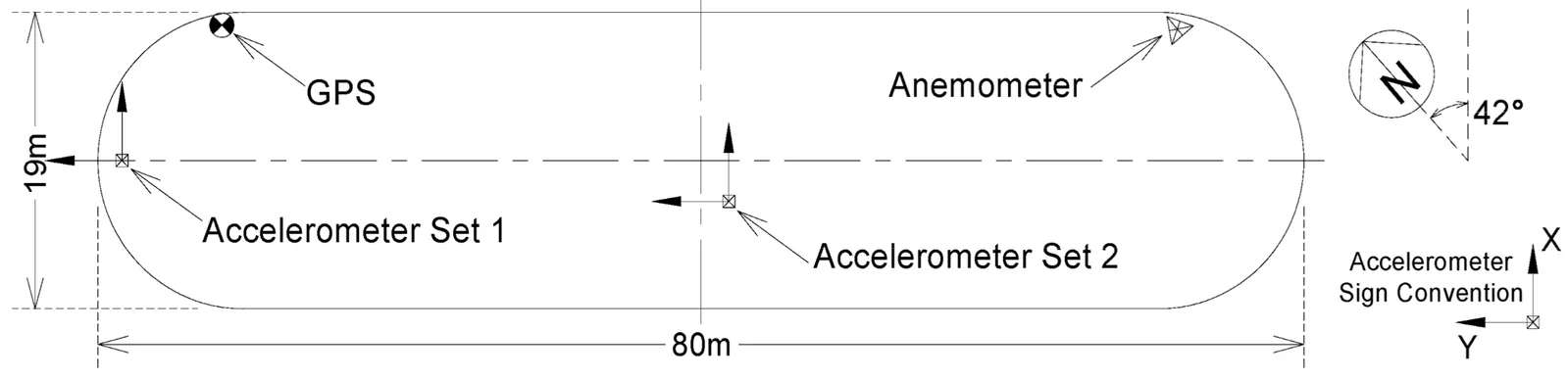
Layout of the monitoring instruments installed on the roof of the super-tall building.

The monitoring system consisted of two TOPCON GB-1000 dual-frequency GNSS receivers, four Honeywell QA-650 accelerometers, and one GILL ultrasonic anemometer. One GNSS receiver was installed at the nearby Hong Kong Polytechnic University as a reference station, whereas the other was installed on the roof of the building as a monitoring station. The baseline length between the two GNSS receivers was approximately 0.84 km. Four accelerometers were installed at different positions on the top floor to monitor the acceleration responses induced by wind excitation. The sampling frequencies of the GNSS receivers and accelerometers were 10 Hz and 20 Hz, respectively. In this study, only the acceleration measurements are used for evaluating the proposed signal processing method, while the other instruments are described to provide the monitoring context. The layout of the monitoring instruments is shown in Figure 15 (reproduced from Yi et al. [30] with permission).

**Figure 16:**
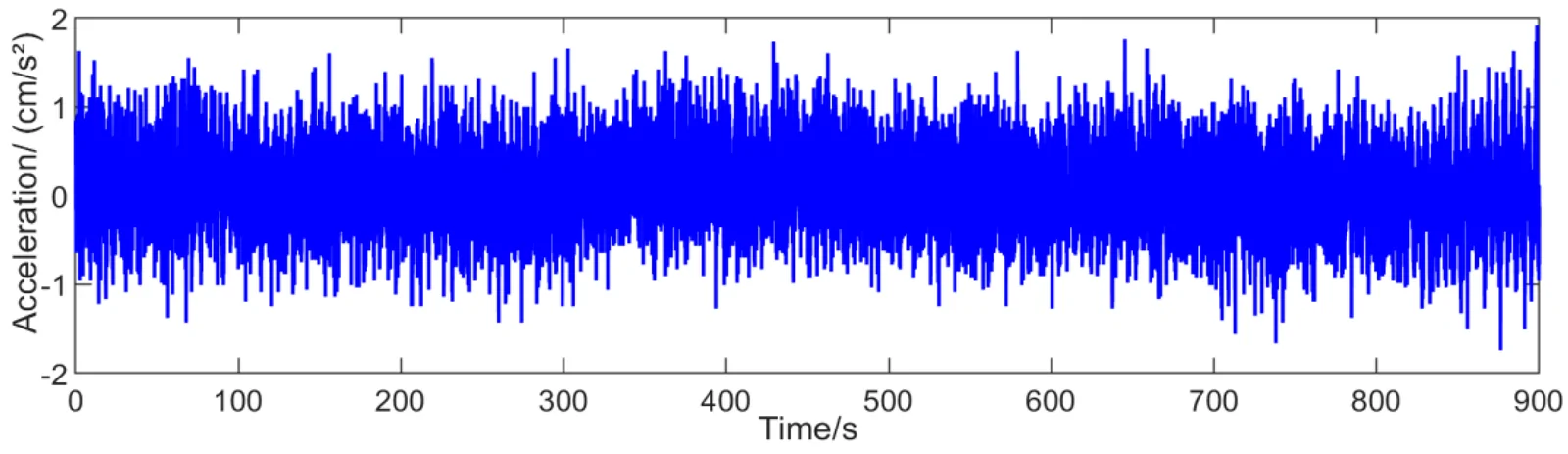
Time-domain waveform of the 15-min acceleration response in the X direction.

The acceleration record measured by the sensor installed at the corner wing of the top floor was selected because the response at this roof-corner location was relatively pronounced during the event and provided a clear record for evaluating the signal-processing method. The data were collected between 10:00 and 11:00 a.m. (UTC + 8) during the passage of a tropical storm. A continuous 15-min X-direction record without missing samples or evident instrumental anomalies was selected to provide sufficient duration for resolving the low-frequency components and characterizing their temporal variations. The interval was selected for signal analysis and was not assumed to represent the period of maximum wind loading. The record was demeaned and detrended to reduce constant bias and low-frequency drift, and its time-domain waveform is shown in Figure 16. The subsequent analysis focuses on the 0.1–2.0 Hz band. Because only one accelerometer location, one direction, and one 15-min interval are analyzed, the record is treated as an application example rather than a spatially comprehensive assessment of the building response.

### 4.2. Vibration Component Extraction and Time-Frequency Characterization

To extract the dominant frequency components, the proposed MTM-eNTFT method was applied to the selected X-direction acceleration record. MTM was first used to obtain stable frequency priors for determining the target frequency regions required for subsequent component extraction. After determining these regions, the MPE-based endpoint extension was applied before NTFT-based component reconstruction to alleviate boundary effects in the finite-length record.

**Figure 17:**
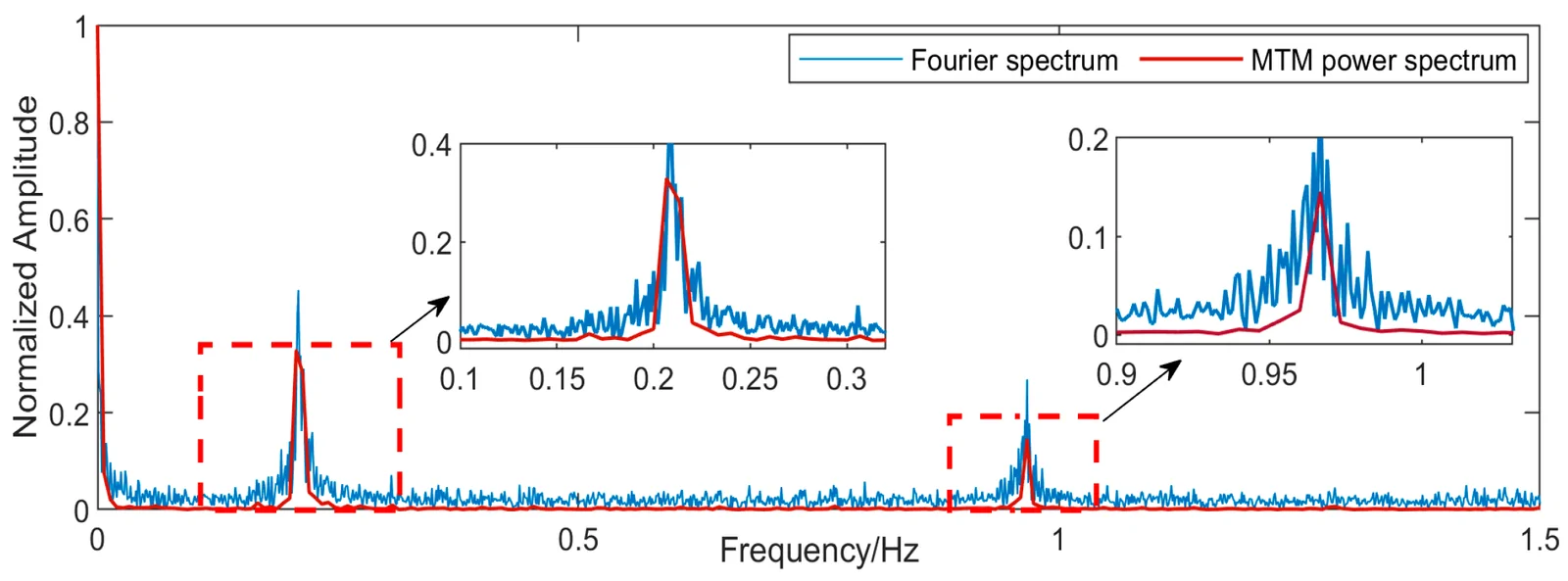
Identification of target frequency regions based on the Fourier spectrum and MTM power spectrum.

Figure 17 compares the conventional Fourier spectrum and the MTM power spectrum. It can be observed that the field monitoring signal contains background noise and random fluctuations. The conventional Fourier spectrum exhibits noticeable local spectral fluctuations, making the boundaries between different frequency components less distinct. In comparison, the MTM power spectrum provides a relatively stable spectral representation, in which the main frequency peaks and their neighboring energy distributions can be identified more clearly.

**Figure 18:**
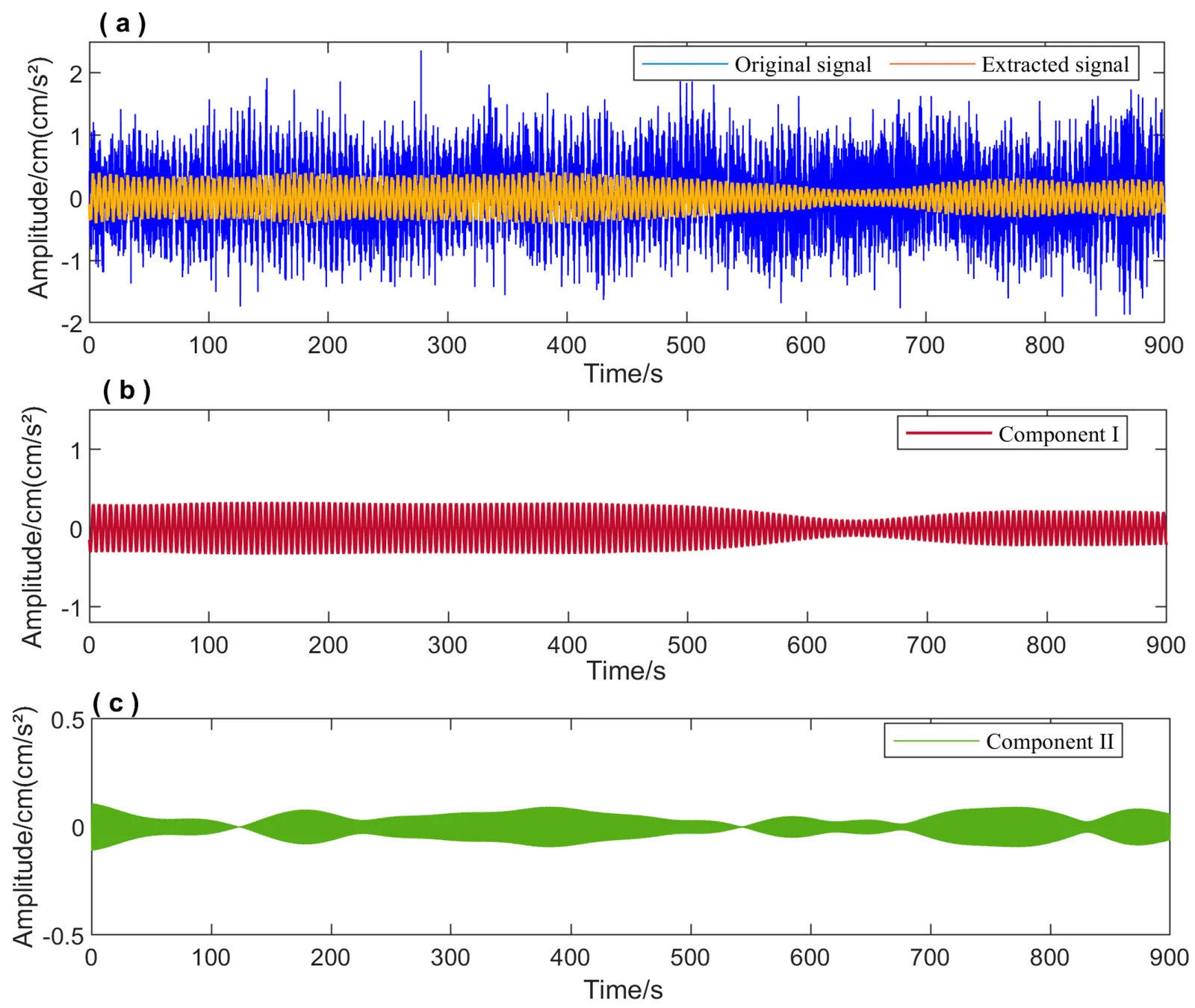
Time-domain waveforms of the measured and extracted acceleration responses: (**a**) original signal; (**b**) extracted component I; (**c**) extracted component II.

**Figure 19:**
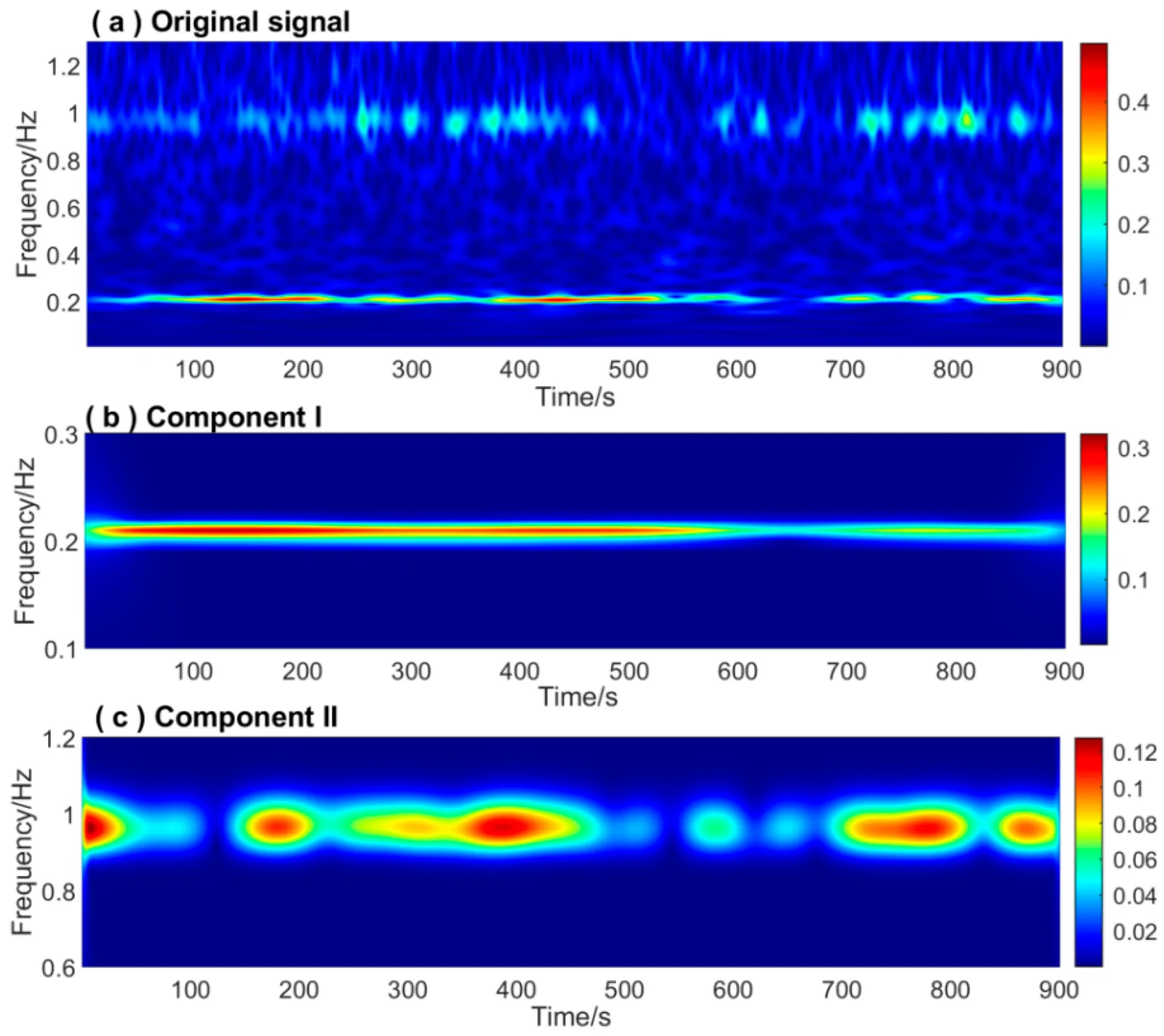
Time-frequency representations of the acceleration responses: (**a**) original signal; (**b**) extracted component I; (**c**) extracted component II.

Based on the peak positions and concentrated energy regions in the MTM spectrum, the target frequency bands are automatically determined following the “local valley” and “energy decay” criteria described in Section 2.3.1. The resulting dominant frequencies and band boundaries are listed in Table 4. These frequency regions are used as frequency-band constraints for the subsequent MTM-eNTFT-based component extraction. Based on Equations (11) and (13), the optimal extension length and its associated MPE value for each signal component were obtained and are listed in Table 4. Endpoint extension details are not shown due to space constraints. MTM-eNTFT was applied to extract the corresponding frequency components from the selected acceleration record. The time-domain waveforms of the original record and the extracted components are shown in Figure 18, and their two-dimensional time-frequency representations are shown in Figure 19. The first component is mainly concentrated around 0.208 Hz and remains observable over most of the selected interval; it is therefore described as the dominant low-frequency component of the record. The second component is mainly distributed around 0.965 Hz and exhibits a more intermittent temporal pattern; it is described as the second dominant frequency component. No specific modal order is assigned to either component in the absence of independent modal evidence.

**Figure 20:**
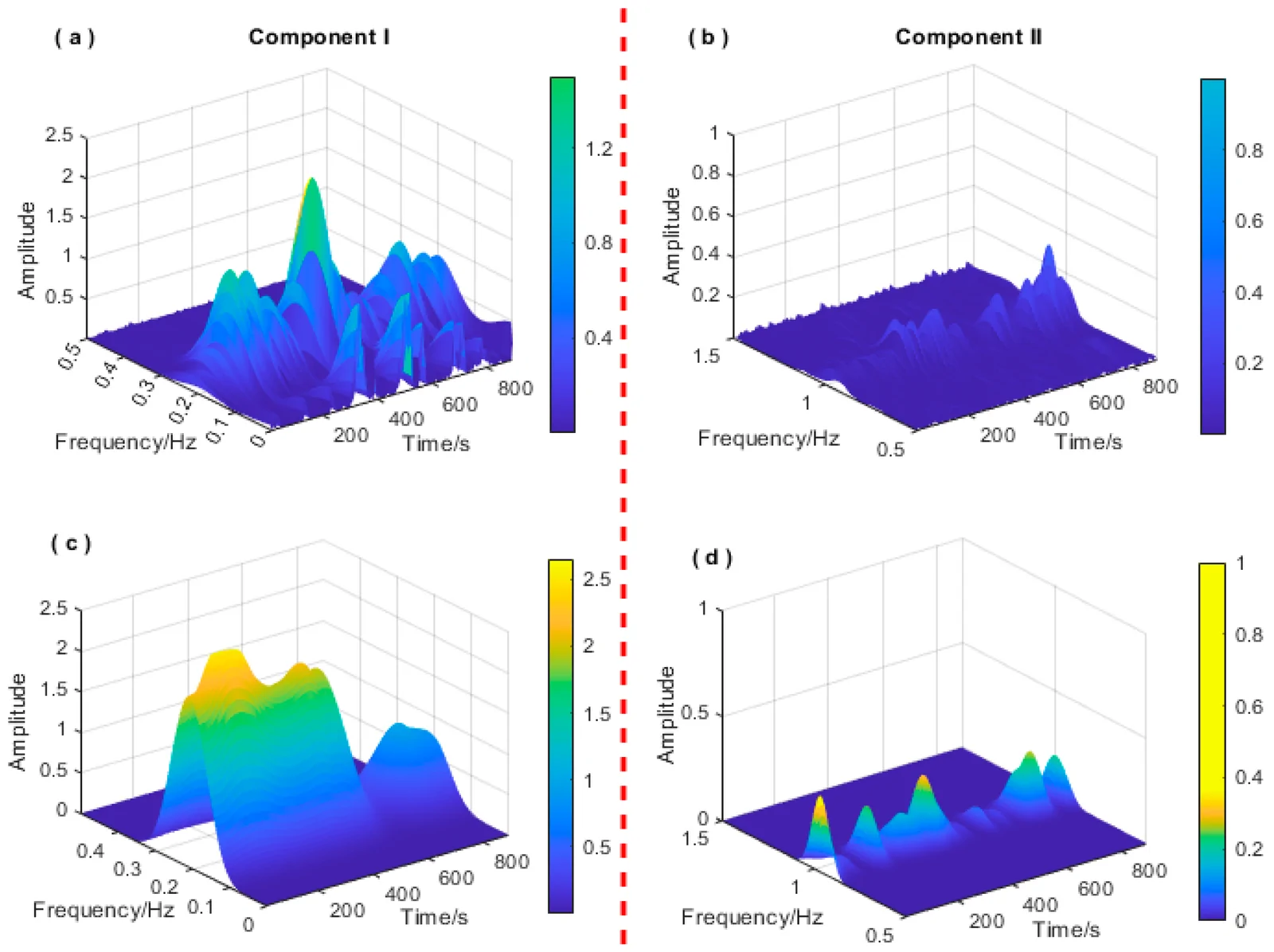
Three-dimensional time-frequency representations before and after MTM-eNTFT-based component extraction: (**a**) original signal component I; (**b**) original signal component II; (**c**) extracted component I; (**d**) extracted component II.

Figure 20 compares the three-dimensional time-frequency representations before and after component extraction. Before processing, the original record contains diffuse background energy and energy distributed outside the selected target-frequency regions, particularly around the higher-frequency component. After MTM-eNTFT extraction, the energy distributions of the two dominant frequency components become more concentrated, while energy outside the selected target-frequency regions is attenuated. The time-varying energy distributions also show an observable increase between approximately 130 and 460 s. This feature may indicate a temporary increase in the measured dynamic response during the selected interval. However, the available single-sensor acceleration record does not establish whether the increase resulted from stronger wind loading, global response amplification, near-resonant behavior, or other factors. These observations are consistent with the time-domain waveforms in Figure 18 and the two-dimensional time-frequency representations in Figure 19.

### 4.3. Discussion of the Field-Case Results

For high-rise buildings, the empirical range of the fundamental frequency can be estimated as:

(24) $$f_{emp}\in \left[\frac{10}{N},\;\frac{20}{N}\right]\;$$

where $f_{emp}$ denotes the empirical range of the fundamental frequency, and N is the number of floors. For the 68-storey building considered in this study, the estimated range is approximately 0.147–0.294 Hz. The component near 0.208 Hz falls within this empirical range. This agreement provides a useful engineering reference for interpreting the extracted low-frequency component, but it does not independently establish its modal order. Accordingly, the two extracted components are described here as dominant frequency components rather than confirmed structural modes.

The increase in the energy of both components between approximately 130 and 460 s is a distinct time-frequency feature of the selected record and is consistent with the nonstationary nature of the measured response. Because the current analysis is based on one acceleration location, one response direction, and a single 15-min interval, it cannot distinguish among variations in wind loading, changes in the global structural response, and other transient or local influences. In particular, the coincidence of the 0.208 Hz component with the empirical frequency range, together with the observed energy increase, is insufficient to confirm near-resonant behavior or global response amplification.

The field application should therefore be regarded as an illustration of component extraction and time-frequency characterization rather than a complete modal assessment of the monitored building. Rigorous physical interpretation would require synchronized wind-speed and wind-direction measurements, spatially distributed structural responses, independent modal-identification results, and comparisons across different environmental and loading conditions. Within the available evidence, the results support the practical applicability of MTM-eNTFT to noisy finite-length structural monitoring records while defining the limits of the conclusions that can be drawn from a single-channel case.

## 5. Conclusions

This study developed an MTM-eNTFT framework for dominant-frequency-component extraction and time-frequency characterization of noisy, multicomponent, finite-length structural response signals. Its main contribution is the integration of multitaper-based target-frequency-region determination, multiscale permutation entropy-guided endpoint extension, and band-limited NTFT reconstruction within a unified procedure. This integration addresses two related challenges: noise-sensitive target-frequency-region selection and endpoint-induced reconstruction distortion.

Under the investigated simulation conditions, the proposed framework improved component reconstruction, noise suppression, and time-domain fidelity, particularly near signal boundaries and during transient intervals. In the field application, two dominant frequency components centered at approximately 0.208 and 0.965 Hz were extracted from the selected 15-min acceleration record. Their energy increased between approximately 130 and 460 s, possibly indicating a temporary increase in the measured dynamic response. Given the single-location, single-direction record, no modal order or specific physical cause is assigned to the extracted components or the observed energy increase.

These results support the applicability of MTM-eNTFT to component extraction and time-frequency characterization of noisy finite-length structural monitoring records. Further validation should incorporate synchronized multi-sensor response and wind measurements together with independent modal-identification results. Future methodological work should also examine adaptive parameter determination, endpoint extension, and transient-component processing under more diverse noise and component-separation conditions.

## Figures and Tables

**Table 1:** MTM-derived frequency and MPE-selected endpoint extension lengths.

Component	Dominant Frequency/Hz	Dominant Period/Samples	MTM-Derived Band/Hz	Edge Effect Length/Samples	Candidate Constraint	$L_{opt}$/Samples	MPE Value
$x_{1}(t)$	0.0488	204.8	0.04~0.06	308	[410,⋯, 819]	410	0.00249
$x_{2}(t)$	0.3003	33.3	0.29~0.31	50	[67,⋯, 999]	766	0.00144
$x_{3}(t)$	0.1489	67.15	0.13~0.17	101	[134,⋯, 940]	604	0.00219

**Table 2:** Ablation comparison of NTFT, MTM-NTFT, and MTM-eNTFT over 30 independent noise trials at an input SNR of −5 dB.

Methods	RMSE	PCC	SNR (dB)	TDF (%)	Edge RMSE
Original input signal	0.6687	0.5116	−5	−77.80	/
NTFT	0.3578 ± 0.0465 [0.3406, 0.3750]	0.3830 ± 0.0390 [0.3684, 0.3976]	0.4342 ± 1.1291 [0.0126, 0.8558]	4.41 ± 12.36 [−0.21, 9.03]	0.3895 ± 0.0530 [0.3697, 0.4093]
MTM-NTFT	0.1085 ± 0.0128 [0.1037, 0.1133]	0.9582 ± 0.0048 [0.9564, 0.9600]	10.7957 ± 1.0241 [10.4132, 11.1782]	71.15 ± 3.40 [69.88, 72.42]	0.1415 ± 0.0190 [0.1345, 0.1485]
MTM-eNTFT	0.0716 ± 0.0045 [0.0699, 0.0733]	0.9833 ± 0.0020 [0.9826, 0.9840]	14.4109 ± 0.5400 [14.2092, 14.6126]	80.96 ± 1.20 [80.51, 81.41]	0.0850 ± 0.0115 [0.0807, 0.0893]

Note: Values are reported as mean ± standard deviation over 30 independent noise trials. Values in brackets denote the two-sided 95% confidence intervals of the mean calculated using Student’s t−distribution. Within each trial, all methods were evaluated using the same noisy realization. Lower RMSE and edge RMSE and higher PCC, output SNR, and TDF indicate better performance.

**Table 3:** Quantitative comparison of CEEMDAN, VMD, SET, and MTM-eNTFT over 30 independent noise trials at an input SNR of −5 dB.

Methods	RMSE	PCC	SNR (dB)	TDF (%)
Original input signal	0.6687	0.5116	−5	−77.80
CEEMDAN	0.1605 ± 0.0095 [0.1571, 0.1639]	0.9075 ± 0.0055 [0.9055, 0.9095]	7.3969 ± 0.5120 [7.2057, 7.5881]	57.25 ± 2.53 [56.3060, 58.1940]
VMD	0.1462 ± 0.0069 [0.1435, 0.1488]	0.9225 ± 0.0072 [0.9198, 0.9252]	8.2169 ± 0.4096 [8.0640, 8.3698]	61.13 ± 1.85 [60.44, 61.82]
SET	0.0830 ± 0.0055 [0.0810, 0.0850]	0.9755 ± 0.0030 [0.9744, 0.9766]	13.1265 ± 0.5758 [12.9115, 13.3415]	77.88 ± 1.46 [77.33, 78.43]
MTM-eNTFT	0.0716 ± 0.0045 [0.0699, 0.0733]	0.9833 ± 0.0020 [0.9826, 0.9840]	14.4109 ± 0.5400 [14.2092, 14.6126]	80.96 ± 1.20 [80.51, 81.41]

Note: All metrics were defined and computed following the protocol described in the footnotes to Table 2.

**Table 4:** Target frequency regions and MPE-selected endpoint extension lengths for signal extraction.

Component	Dominant Frequency/Hz	Dominant Period/Samples	MTM-Derived Band/Hz	Edge Effect Length/Samples	Candidate Constraint	$L_{opt}$/Samples	MPE Value
I	0.2148	93.09	0.18~0.23	140	[186,⋯, 5958]	1676	0.00126
II	0.9656	20.71	0.94~0.98	32	[41,⋯, 5986]	2030	0.00219

## Data Availability

Data available on request from the authors.
